# Crossover Localisation Is Regulated by the Neddylation Posttranslational Regulatory Pathway

**DOI:** 10.1371/journal.pbio.1001930

**Published:** 2014-08-12

**Authors:** Marina Tagliaro Jahns, Daniel Vezon, Aurélie Chambon, Lucie Pereira, Matthieu Falque, Olivier C. Martin, Liudmila Chelysheva, Mathilde Grelon

**Affiliations:** 1 INRA, Institut Jean-Pierre Bourgin, UMR 1318, ERL CNRS 3559, Saclay Plant Sciences, RD10, Versailles, France; 2 AgroParisTech, Institut Jean-Pierre Bourgin, UMR 1318, ERL CNRS 3559, Saclay Plant Sciences, RD10, Versailles, France; 3 Institut National de la Recherche Agronomique, Unité Mixte de Recherche de Génétique Végétale, Université Paris-Sud, Gif-sur-Yvette, France; National Cancer Institute, United States of America

## Abstract

Crossovers (COs) are at the origin of genetic variability, occurring across successive generations, and they are also essential for the correct segregation of chromosomes during meiosis. Their number and position are precisely controlled, however the mechanisms underlying these controls are poorly understood. Neddylation/rubylation is a regulatory pathway of posttranslational protein modification that is required for numerous cellular processes in eukaryotes, but has not yet been linked to homologous recombination. In a screen for meiotic recombination-defective mutants, we identified several *axr1* alleles, disrupting the gene encoding the E1 enzyme of the neddylation complex in *Arabidopsis*. Using genetic and cytological approaches we found that *axr1* mutants are characterised by a shortage in bivalent formation correlated with strong synapsis defects. We determined that the bivalent shortage in *axr1* is not due to a general decrease in CO formation but rather due to a mislocalisation of class I COs. In *axr1*, as in wild type, COs are still under the control of the ZMM group of proteins. However, in contrast to wild type, they tend to cluster together and no longer follow the obligatory CO rule. Lastly, we showed that this deregulation of CO localisation is likely to be mediated by the activity of a cullin 4 RING ligase, known to be involved in DNA damage sensing during somatic DNA repair and mouse spermatogenesis. In conclusion, we provide evidence that the neddylation/rubylation pathway of protein modification is a key regulator of meiotic recombination. We propose that rather than regulating the number of recombination events, this pathway regulates their localisation, through the activation of cullin 4 RING ligase complexes. Possible targets for these ligases are discussed.

## Abstract 2

A genetic study finds the neddylation pathway (known to-date for post-translational protein modification) is involved in regulating crossover localization but not crossover number during meiosis in *Arabidopsis*.

## Author Summary

During meiosis, two successive chromosomal divisions follow a single S phase, resulting in the formation of four haploid cells, each with half of the parental genetic material. This reduction in chromosome number occurs during the first meiotic division, when homologous chromosomes (paternal and maternal) are separated from each other. For this to happen, homologous chromosomes associate in structures called bivalents, where each chromosome is linked to its homologue by a point of contact known as chiasmata. These chiasmata reflect the formation of crossovers (COs), one of the manifestations of the exchange of genetic material occurring during homologous recombination. CO number varies little at around two per chromosome pair, and they tend to be evenly spaced on chromosomes. Thus, CO number and distribution are very tightly controlled. However, the mechanisms underlying these controls are very poorly understood. In this study, we identified a regulatory pathway of meiotic recombination. We show that this pathway does not regulate the amount of recombination events per se, but instead controls their localisation, as when it is defective, CO events cluster together in a few regions of the genome, leading to bivalent shortage and progeny aneuploidy with incorrect numbers of chromosomes. This regulatory pathway is a posttranslational protein modification system called neddylation (or rubylation in plants), known to be required for numerous cellular processes in eukaryotes. We identify an enzyme of the neddylation complex as a major regulator of meiotic recombination in *Arabidopsis* and show that this process may be also conserved in mammals.

## Introduction

Meiosis is a modified cell cycle where two rounds of chromosome segregation follow a single S phase, resulting in the production of haploid gametes. Recombination is a key step in meiosis I, as it results in genetic crossover (CO) formation, which establishes physical links between the homologues cytologically visible as chiasmata [1],[2]. In most species, each chromosome pair has at least one CO (referred to as the obligatory CO), which is required to hold the homologues together during the first meiotic division, ensuring their correct segregation. In most organisms, homologues that lack a CO often segregate improperly, leading to the formation of aneuploid gametes [3]. Meiotic recombination can also lead to gene conversion not associated with COs (NCOs) [4].

Meiotic recombination is initiated by the induction of DNA double-strand breaks (DSBs) catalysed by SPO11 [5]. DSBs are then resected by exonucleases to generate 3′ single-stranded DNA molecules (ssDNA). In the subsequent step, RecA homologues RAD51 and DMC1 assemble on the ssDNA to form nucleoprotein filaments. These filaments search for homologous sequences and trigger single-strand invasions [6] to generate displacement loop (D-loop) recombination intermediates [7]. Depending on the way these D-loop intermediates are processed, different recombination products can be formed. For example, capture of the second DSB end leads to the formation of a double Holliday junction that can be resolved to generate either a non-CO (NCO) or a CO [8]–[10]. Alternatively, NCOs can also be formed when a single strand end is displaced after priming a limited amount of DNA synthesis, annealing with the other DSB end in a process called synthesis-dependent strand annealing (SDSA) [11].

In most organisms, when multiple COs occur on the same chromosome, they are distributed nonrandomly: One CO prevents other COs from occurring close by, in a distance-dependent manner. This phenomenon results in COs being more evenly spaced along chromosomes than would be expected if they occurred randomly. The term used to describe this phenomenon is CO interference [12],[13]. In budding yeast, two kinds of COs are known to coexist: class I COs, which are interference-sensitive COs and whose formation depends on the ZMM proteins (Zip1, Zip2, Zip3, Zip4, Msh4, Msh5 and Mer3) in addition to Mlh1 and Mlh3, and class II COs, which are not subject to interference and depend on Mus81 and Eme1/Mms4 [10]. *Arabidopsis thaliana*, like yeast and mammals, has two recombination pathways: one that exhibits CO interference and another one that does not [14]–[20]. In *A. thaliana*, disruption of genes acting in the interference-sensitive pathway causes a loss of approximately 85% of COs [21]. In addition, there is evidence that the *MUS81* gene accounts for some, but not all, of the 15% *MSH4*-independent COs, suggesting that MUS81 is involved in a secondary subset of meiotic COs that are interference insensitive [14],[22]. Very little information is available on the mechanisms controlling interference and the number and distribution of COs during meiosis in general [23],[24].

Eukaryotes possess a highly conserved mechanism to control protein degradation mediated by the action of the ubiquitin (Ub) proteasome system (UPS) [25]. In this system, E3 Ub ligases are required to ubiquitylate specific protein targets. Cullin RING ligases (CRLs) are the largest class of E3 ligases. Several mechanisms control CRL activity: It can be activated by covalent attachment of the Ub-like protein NEDD8/RUB (a process called neddylation or rubylation) [26],[27] or inhibited by the COP9 signalosome-directed deneddylation [28]. Neddylation/rubylation has been shown to play a crucial role in processes such as morphogenesis in mice [29], cell division in budding yeast [30], embryogenesis in *C. elegans* [31], meiosis to mitosis transition in *C. elegans* [32], and response to various plant hormones [33],[34] including auxin [35]–[38]. However, neddylation/rubylation had not been connected to homologous recombination (HR).

Cullin RING Ligase 4 (CRL4) is associated with DNA repair in plants and humans; the DDB1-CUL4A^DDB2^ E3 ligase initiates nucleotide excision repair (NER) by recognizing damaged chromatin with concomitant ubiquitylation of core histones at the lesion site [39]–[41]. Additionally, CUL4A plays a role in meiotic recombination and spermatogenesis in mice [42],[43]. Inactivation of *cul4a* affected male fertility, with increased death of pachytene/diplotene cells and defects in MLH1 dissociation from the SCs.

Here we show that the E1 enzyme of the neddylation complex, AXR1, is a major regulator of meiotic recombination in *Arabidopsis*. In *axr1* mutants, the average number of meiotic COs is unchanged; they are still under the control of the ZMM proteins, but they tend to cluster together and no longer follow the obligatory CO rule. We were able to show that this recombination defect is correlated with strong synapsis defects. In addition, we found that this deregulation of CO localisation is likely mediated by a CRL4.

## Results

### The axr1 Mutants Are Meiosis-Defective

**Figure 1:**
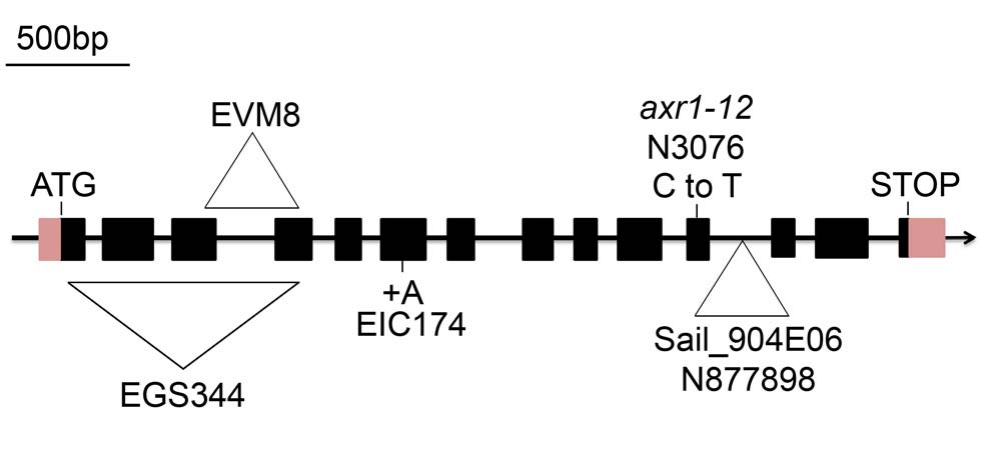
The *AXR1* gene and *axr1* mutations. The arrow indicates the orientation of the open reading frame. Exons are shown as boxes (pink, UTR; black, CDS). In the EGS344 mutant, a large deletion associated with an insertion of the exogenous *Agrobaterium* Ti plasmid disrupts the *AXR1* gene from nucleotide 91 (40 bp 5′ to the ATG). In the EVM8 mutant, an in-frame deletion of 312 bp between exons 3 and 4 generates a 20 aa truncated protein. In EIC174, a single nucleotide insertion (A) in exon 6 (position 1364 of the genomic sequence, corresponding to nt 688 in the cDNA) leads to a premature stop codon (a 222 aa protein is produced instead of the 540 aa protein in wild type). In *axr1-12*, corresponding to the N3076 line, a single C-T nucleotide substitution at position 1295 of the cDNA leads to a premature stop codon (415 aa instead of 540), as described by Leyser et al. [44]. In N877898, corresponding to the Sail_904E06 line, a T-DNA insertion occurred in intron 11. References used for this figure are Tair accession 4010763662 for the genomic sequence and Tair accession 4010730885 for the cDNA sequence.

In the process of screening *A. thaliana* T-DNA (*Agrobacterium tumefaciens* transferred DNA) insertional lines for meiotic defects, we isolated three mutants [EGS344, EIC174, and EVM8 (Ws-4 strain); Figure 1 and Figure S1] allelic for disruption in At1g05180, the *AXR1* gene, previously shown to encode the E1 enzyme of the *Arabidopsis* neddylation complex [44]. Another insertion line in At1g05180 available in the public collection (http://signal.salk.edu/) Sail_904E06 (N877898, Col-0 strain) and the historical *axr1* allele (*axr1-12*/N3076, Col-0 ecotype [44], with a single nucleotide substitution in exon 11 of At1g05180) were also included in this study (Figure 1).

**Figure 2:**
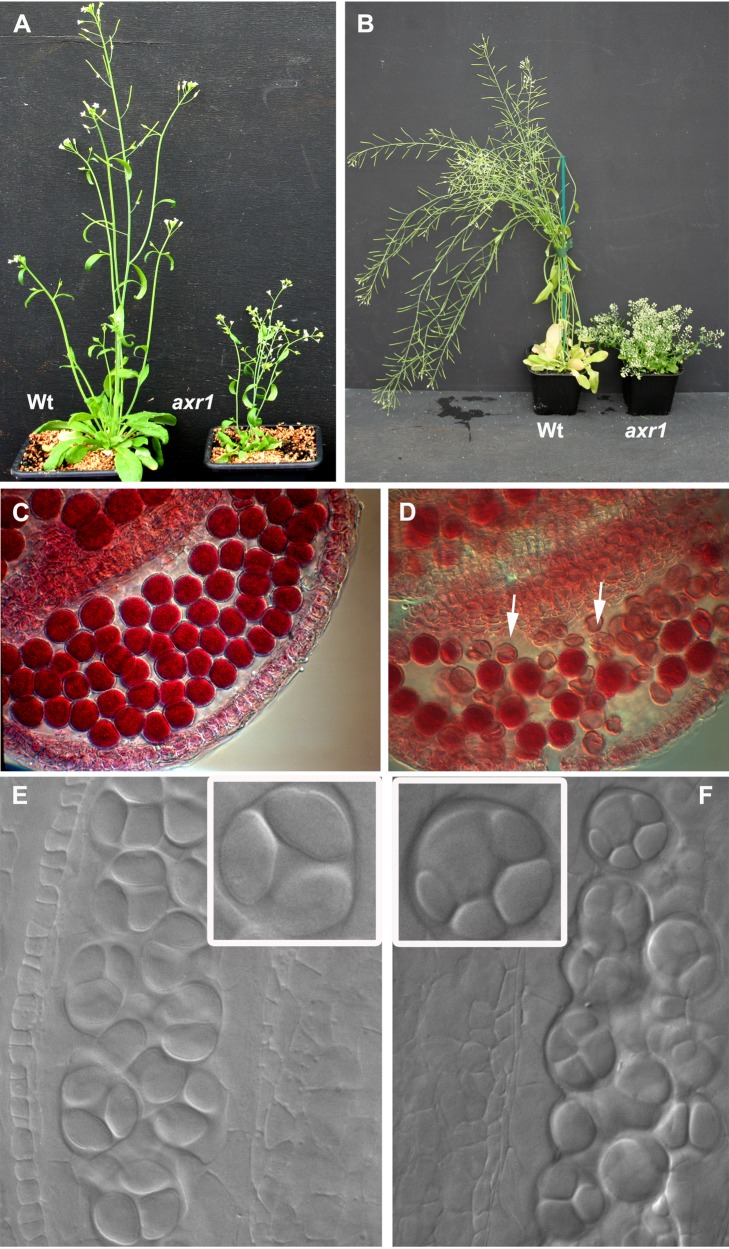
*axr1* developmental defects. Five- (A) or nine- (B) week-old wild-type (wt) or *axr1* (N877898) mutant plants. *axr1* mutants are dwarfed, strongly branched, and have short siliques. Alexander staining (C–D) reveals round pollen grains, with a red cytoplasm reflecting viable male gametophytes in wild type (C), whereas *axr1* (N877898) anthers (D) contain a mixture of viable and dead (uncoloured, arrows) pollen grains. DIC microscopy of male meiosis products (E and F) reveals tetrads of microspores in wild-type (E) and unbalanced tetrads or polyads in *axr1* (F, N877898).

The mutant plants all show the same vegetative phenotypes as previously described for *axr1* mutants: They are dwarfed, excessively branched, with small rosettes and crinkled leaves (shown for N877898 in Figure 2A–B and in Figure S2 for the other alleles) [45],[46]. They also have small flowers and short fruits, indicating fertility defects (Figure S2).

We examined the reproductive development of these mutants and found that all alleles showed a high level of male and female gametophyte abortion [shown for N8779898 male gametophytes (pollen grains) in Figure 2D]. In plants, male gametogenesis occurs in the anthers where groups of meiocytes undergo meiosis synchronously, each producing four haploid cells (called microspores). The four products of each meiosis remain temporally encased in a common callose wall, forming tetrads of microspores that can be visualised after tissue clearing (Figure 2E). Each microspore is then released from its tetrad and continues to develop into a mature pollen grain (the male gametophyte) containing the male gametes. Study of the early stages of pollen development in *axr1* revealed the presence of abnormal meiotic products. Instead of the regular tetrahedral structure observed in the wild-type, asymmetric tetrads (containing four daughter cells of unequal size) or “polyads” (containing more than four products) were observed (Figure 2F), suggesting that the meiotic program is disrupted in these mutants.

**Figure 3:**
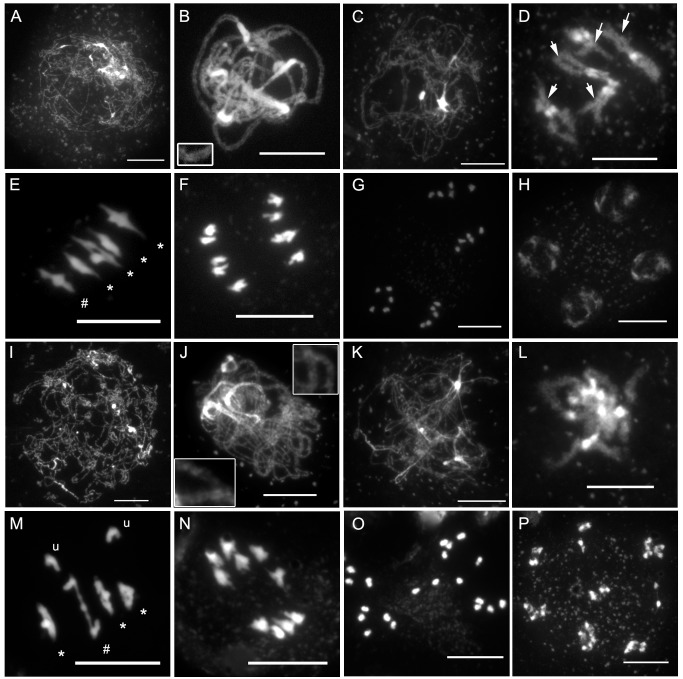
*axr1* mutants show normal meiotic progression but reduced bivalent formation at metaphase I. DAPI staining of meiotic chromosomes in wild type (A–H) and *axr1* (N877898, I–P). At the onset of meiotic prophase I (A and I), chromosomes can be identified. Chromosome alignment and synapsis then proceeds, leading eventually to the pachytene stage in wild type (B), where homologous chromosomes are synapsed along their entire length. This association can be observed in *axr1* (J, enlarged regions) but remains partial. Then, the SC disappears at diplotene (C and K), condensation proceeds, and bivalents can be identified in wild type at diakinesis (D), but this stage is rarely observed in *axr1* (L). At metaphase I, the five *Arabidopsis* bivalents can be identified in wild type (E), segregating at anaphase I (F). In *axr1*, a mixture of bivalents and univalents are observed (M), leading to subsequent improper segregation at anaphase I (N). Sister chromatids segregate at meiosis II (G and O), leading to balanced tetrads in wild type (H), unbalanced tetrads (not shown) or polyads in *axr1* (P). At metaphase I, univalents (u) can be distinguished from ring bivalents (where a chiasma occurred in each of the two chromosome arms, *) and from rod bivalents (where only one chromosome arm shows a chiasma, #). Arrows in (D) indicate some of the chiasma-containing arms. Bar, 10 µm.

To confirm that the reduced fertility was caused by a defect in meiosis, we investigated male meiosis via chromosome spreading and DAPI (4′,6-diamidino-2-phenylindole) staining (Figure 3). During wild-type meiotic prophase I (Figure 3A–D), DNA fibres of each sister chromatid are organised as chromatin loops connected to a common protein axis (the axial element [AE]) [47]. When chromosomes start to condense at leptotene, they become visible as threads (Figure 3A). At this stage, meiotic recombination is initiated by the formation of a large number of DNA DSBs (not shown). HR repairs these breaks concomitantly with the progression of synapsis, the close association of the homologous chromosome axes through the polymerisation of the central element (CE) of the synaptonemal complex (SC). Synapsis begins at zygotene (not shown) and is complete by pachytene, when complete alignment of homologous pairs can be detected in DAPI-stained chromosomes (Figure 3B). DNA repair and recombination are thought to be achieved during pachytene, yielding at least one CO per homologous chromosome pair. At diplotene (Figure 3C), when the CE of the SC is depolymerised, the homologous chromosomes are therefore connected to each other by COs in which chromatids from homologous chromosomes have been exchanged. These connections between homologous chromosomes become apparent only at diakinesis (Figure 3D, arrows), when chromosomes are sufficiently condensed. At this stage in *Arabidopsis*, chiasmata (the cytological manifestations of COs) cannot be scored precisely, but chiasma-carrying chromosome arms can sometimes be identified based on bivalent appearance (see Figure 3D, arrows). Next, condensation proceeds and, at metaphase I, the five *Arabidopsis* bivalents are easily distinguishable, aligned on the metaphase plate (Figure 3E). During anaphase I, sister chromatid cohesion is released from chromosome arms, allowing homologous chromosomes to segregate to the two opposite cellular poles (Figure 3F). The second meiotic division then separates the sister chromatids, generating four pools of five chromosomes (Figure 3G and 3H), which gives rise to the tetrads of four spores (Figure 2E).

In *A. thaliana axr1* mutants, the leptotene and zygotene stages appeared similar to those in the wild type. However, no pachytene cells were identified in the 457 meiocytes analysed, in contrast to wild type, where this stage is present in approximately 35% of the cells (*n* = 334). Instead, we observed pachytene-like stages, with only partial chromosome alignment (Figure 3J). This suggests that *axr1* is defective in synapsis. Diplotene cells were indistinguishable from those in the wild type (Figure 3K). Then, chromosome condensation could be followed until metaphase I, although diakinesis stages were rarely observed (1% of all stage cells, *n* = 457 for N877898, 12% in wt, *n* = 334) (Figure 3L).

**Figure 4:**
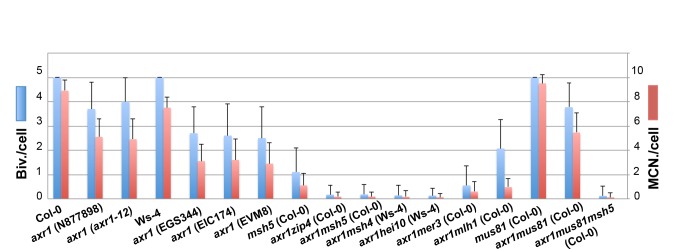
COs in *axr1* are largely ZMM-dependent. For each *axr1* allele and for their respective wild-type strains (Ws-4 for EGS344, EIC174, EVM8, and Col-0 for N8777898 and N3076), and for a combination of multiple mutants, the level of bivalent formation as well as the MCN per cell were measured. For multiple mutant analyses, the N877898 allele was used in a Col-0 background and EGS344 in a Ws-4 background. The complete dataset can be found in Table S1.

At wild-type metaphase I, the five typical *Arabidopsis* bivalents could be observed aligned on the metaphase plate (Figure 3E). Each bivalent was composed of two homologous chromosomes connected by chiasmata either on one chromosome arm (rod bivalent, Figure 3E#) or on both pairs of chromosome arms (ring bivalent, Figure 3E*). Chiasma numbers could therefore be estimated based on the bivalent structure. However, because multiple COs on a single arm cannot be cytologically differentiated from single COs, these estimates only correspond to a minimum chiasma number (MCN; Figure 4, Table S1).

In *axr1* mutants, we observed reduced bivalent formation, and instead of five bivalents, a mixture of bivalents and univalents could be identified (Figure 3M). The reduction in bivalent formation resulted in chromosome mis-segregation during subsequent anaphase I (Figure 3N), whereas the second meiotic division separated sister chromatids (Figure 3O), giving rise to a variable number of daughter cells containing aberrant numbers of chromosomes (Figure 3P).

We quantified the decrease in bivalent formation as well as the MCN at metaphase I from all *axr1* mutants and their respective wild-type accessions (Figure 4, Table S1). On average, *axr1* mutants had 78% of the wild-type number of bivalents for the Col-0 background and 52% for the Ws background. In terms of the chiasma number, *axr1* mutants displayed a residual level of 56% and 41% of the wild-type levels for Col-0 and Ws strains, respectively (Figure 4). Within a single ecotype (Col-0 or Ws), all alleles were statistically different from the wild type but not different from each other. Finally, when the partitioning of the residual chiasmata in *axr1* was analysed, we observed that a large proportion of metaphase I cells showed both ring bivalents (at least two chiasmata) together with univalents (no chiasma) (42% of the N877898 cells, *n* = 47), showing that in *axr1*, the obligatory CO is lost.

**Figure 5:**
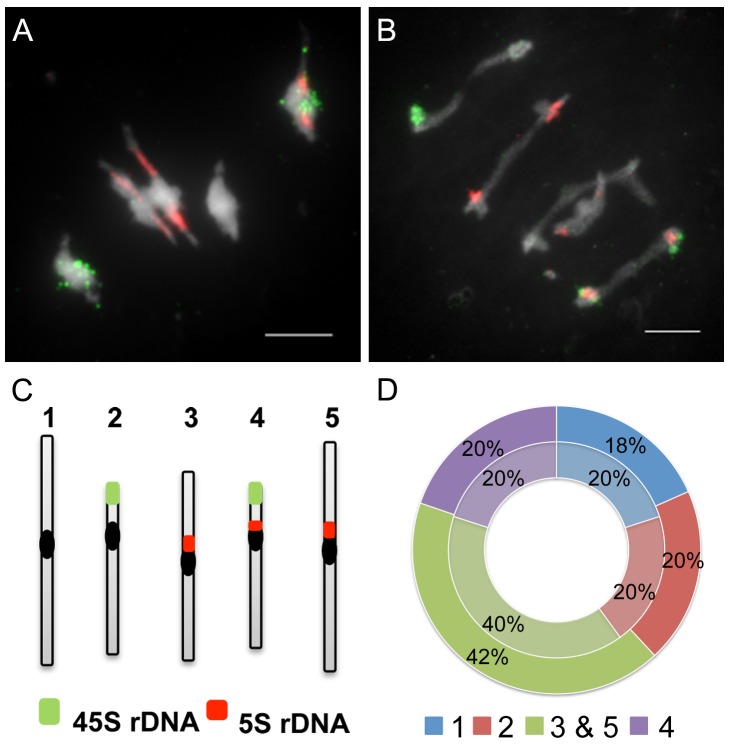
Bivalent shortage has a similar effect on each pair of chromosomes. Fluorescent in situ hybridisation (FISH) on metaphase I cells was performed with probes directed against the 45S (green) and the 5S (red) rDNA, which allow the identification of chromosomes 1 (unlabelled), 2 (green labelled), and 4 (green and red labelled), whereas chromosomes 3 and 5 cannot be distinguished (red labelled). In wild type, each chromosome pair represents 20% of the total number of bivalents (A and D, centre circle, in light, *n* = 21 cells). In *axr1* (B and D, N877898 allele, external circle, *n* = 28), the proportion of each bivalent pair is the same as in wild type. Bar = 5 µm.

To further analyse the bivalent shortage observed in *axr1*, we used fluorescence *in situ* hybridization (FISH) analyses on PMCs. Metaphase I chromosomes were labelled with probes for the 45S and 5S rDNA repeats, allowing specific identification of chromosomes 1, 2, and 4 (Figure 5). Chromosomes 3 and 5 could not be discriminated from each other with these probes and were pooled. First, we observed that in *axr1* as in wild type, bivalents were always formed between homologous chromosomes (*n* = 147 bivalents for *axr1*, *n* = 165 for wt). Then, we considered each bivalent individually and determined which pair of chromosomes was involved in its formation. As shown in Figure 5D, in *axr1*, as in the wild type, each pair of chromosomes was equally involved in bivalent formation, showing that the decrease in bivalent formation observed in *axr1* affected all chromosomes in the same way.

### axr1 COs Are ZMM-Dependent

In wild-type *Arabidopsis*, the majority of COs (85%–90%, depending on the genetic background Col-0 versus Ws-4) depend on the ZMM proteins (MSH4, MSH5, MER3, ZIP4, SHOC1/ZIP2, HEI10, and PTD) as well as on MLH1 and MLH3 [21],[48], whereas MUS81 is responsible for 10%–15% of the remaining COs [14],[22].

We measured bivalent formation frequencies and the chiasma frequencies in various genetic combinations compared to the single *axr1* mutant (Figure 4, Table S1). For all the *zmmaxr1* double mutants (except *mer3axr1*) and regardless of strain (Col-0 versus Ws-4), the level of bivalent formation was reduced by more than 95% with hardly any bivalents observed (from 0.13 to 0.18 bivalent per cell; Table S1), showing that almost all the COs in *axr1* are ZMM-dependent.

We also analysed the bivalent frequency in the *axr1mus81* double mutant, which was the same as for the *axr1* single mutant (3.77±1.03 against 3.75±1.12; *p* = 0.9) (Figure 4). We then quantified bivalent frequency in the *axr1msh5mus81* triple mutant and observed, as expected, a dramatic decrease in bivalent formation compared to *axr1mus81* (Figure 4). No difference could be detected between the *axr1msh5mus81* triple mutant and the *axr1msh5* double mutant (*p* = 0.2). These results show that CO formation in *axr1* mutants is almost exclusively dependent on ZMM proteins, whereas the MUS81 pathway plays only a limited role, if any.

### Class I COs Are Mislocalised in the axr1 Mutant

**Figure 6:**
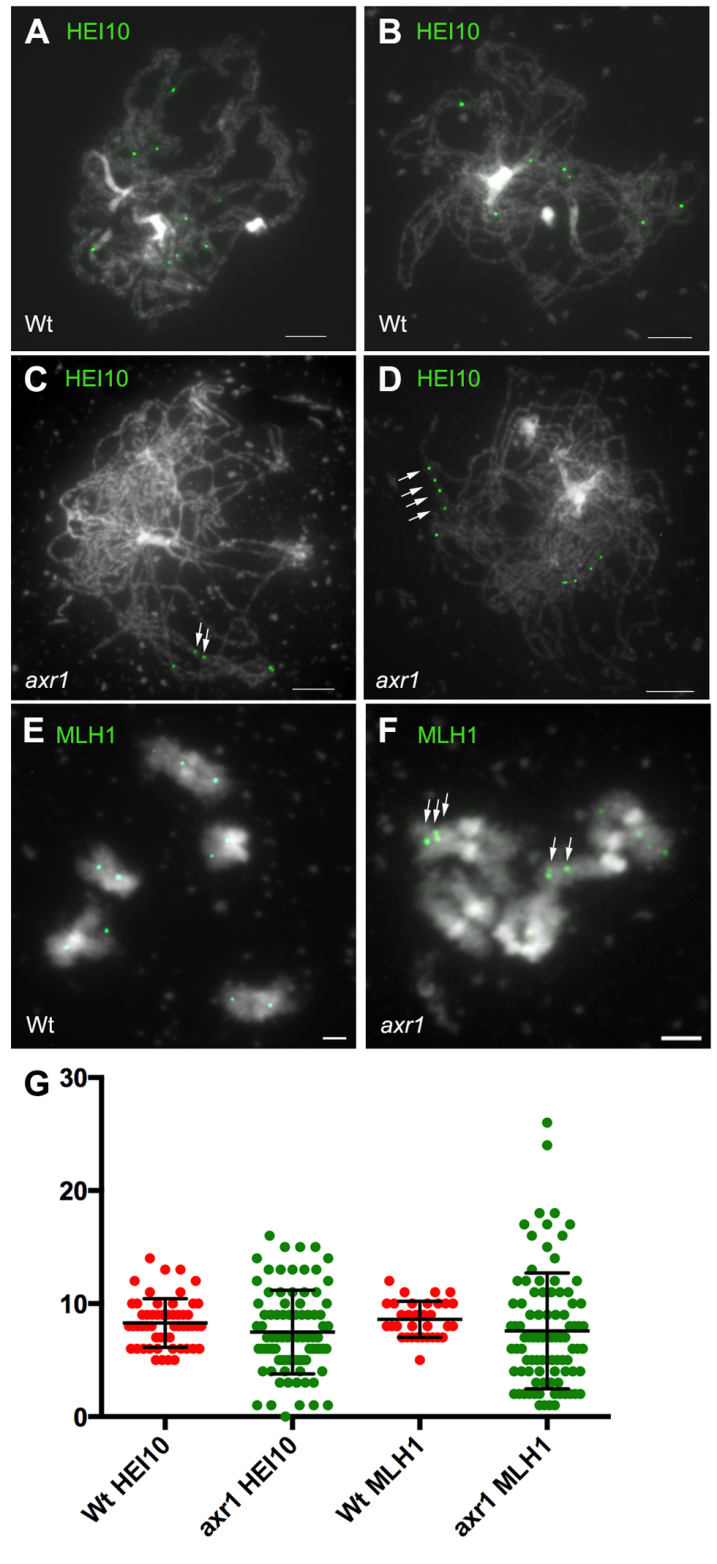
The average number of class I COs is similar in wild type and *axr1*. HEI10 or MLH1 was immunolocalised on acetic acid spread chromosomes from wild-type (A, B, and E, Col-0) or *axr1* (C, D, and F) meiocytes from late pachytene to diakinesis. In *axr1* (N877898 allele), the average number of HEI10 or MLH1 foci per cell is similar to that in wild type (G). Bar  =  5 µM.

To further analyse recombination events in *axr1*, we immunolabelled chromosomes with antibodies directed against HEI10 and MLH1, two markers of class I COs in *Arabidopsis* [48],[49]. MLH1 foci can be seen from late pachytene to diakinesis [49], whereas HEI10 is first loaded early during prophase on a large number of sites forming foci of different sizes on chromosomes. A limited number of these foci then remain (Figure 6A and B) at sites that correspond to class I COs where they co-localise with MLH1 until the end of prophase [48]. We therefore counted HEI10 and MLH1 foci in late pachytene and diplotene cells in wild type and *axr1*. Surprisingly, the average foci number per cell was not different between wild type and *axr1*, for either HEI10 (8.30±0.29, *n* = 54 and 7.49±0.40, *n* = 84, *p* = 0.15) or MLH1 (8.61±0.29, *n* = 33 and 7.58±0.54, *n* = 91, respectively, *p* = 0.263). In addition, we confirmed that these foci localise to chiasma-containing arms at diakinesis (Figure 6E and F and Figure S3), showing that they are likely to mark CO sites in *axr1* as in wild type [49]. We also observed that there was higher variability in the numbers of HEI10 and MLH1 foci in *axr1* than in wild type (Figure 6G), with the coefficient of variation (standard deviation divided by the mean) varying from 26% (HEI10, wt) to 50% (HEI10, *axr1*) or from 19% (MLH1, wt) to 68% (MLH1, *axr1*).

**Figure 7:**
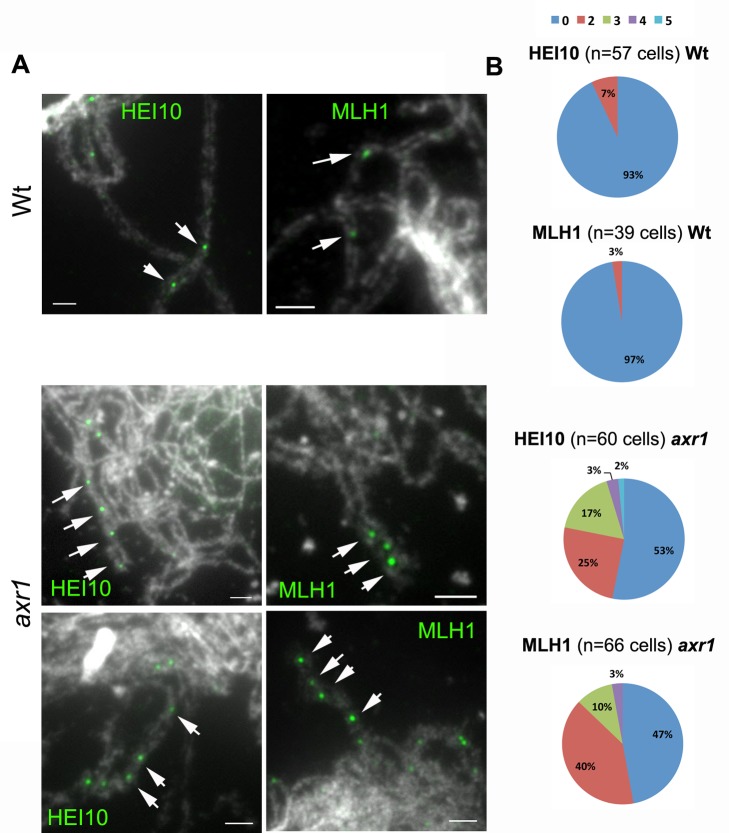
Class I COs tend to cluster in *axr1*. (A) Examples of adjacent HEI10 or MLH1 foci in wild-type (Wt, Col-0) and in *axr1* (N877898 allele) acetic acid spread meiotic chromosomes. (B) Proportion of pachytene and diplotene cells where adjacent foci were observed on the same chromosome axis pair (Wt, wild type; *axr1*, N877898 allele) (0, no evidence of adjacent foci; 2, two adjacent foci, etc.). Some of these situations are indicated by arrows in panel A. Bar  =  5 µM.

Another striking feature of *axr1* was the frequent occurrence at the pachytene-like and diplotene stages of portions of paired chromosome axes where adjacent HEI10 and MLH1 foci could be seen (Figure 6C, D, arrows and Figure 7A, arrows). Forty-seven percent (HEI10, *n* = 60) or 53% (MLH1, *n* = 66) of the cells had at least two foci localised on the same portion of a chromosome axis, whereas in wild type, this scenario occurred only in 7% (HEI10, *n* = 57) or 3% of the cells (MLH1, *n* = 39) (Figure 7B). In addition, although we never observed more than two adjacent foci in wild type, we observed 22% (HEI10) and 13% (MLH1) of the cells with more than two adjacent foci, with a maximum of five adjacent HEI10 foci observed in *axr1* (Figure 7B). Therefore, although the average level of class I COs is the same in *axr1* and in wild type (Figure 6G), these class I COs tend to cluster together in at least 50% of the *axr1* cells.

We then estimated the scale at which this clustering arises. The distance between clustered foci was measured and compared to the total length of chromosome axis. The distance between two adjacent foci was on average 1/400 of the total axis length of a cell, ranging from 1/1600 of the genome to a maximum of 1/90 of the genome (Figure S4A). Extrapolated in DNA distance, with the additional assumption that genome condensation is homogeneous, the distance between two adjacent foci in a cluster is therefore expected to vary from 150 kb to 3,000 kb, with an average of 625 kb. We also observed that the distance between two adjacent foci does not vary significantly in clusters with exactly two foci compared with clusters with more than two foci. As a consequence, cluster size increases proportionally with the number of foci present in the cluster (Figure S4B). The size of the clusters was on average 1/200 of the genome for HEI10 foci (*n* = 14, 1,200 kb) and 1/300 for MLH1 (*n* = 21, 800 kb).

Finally, we examined whether the clustered foci displayed interference, as might be expected for class I COs. We thus considered the hypothesis *H_0_* that the foci in clusters are not subject to interference. The test was based on the distribution of distances between adjacent foci, specifically using the coefficient of variation for the statistical test and comparing to 10^5^ simulations under *H_0_* (see Materials and Methods). For the clusters of three or more foci (Table S2), we rejected the *H_0_* hypothesis of no interference for MLH1 foci (*p* = 0.0024 based on seven clusters), for HEI10 foci (*p* = 0.0028 based on six clusters), and when pooling the MLH1 and HEI10 data (*p* = 2.4×10^−5^ based on 13 clusters). Specifically, inside clusters, MLH1 and HEI10 foci are more evenly distributed than at random, showing that COs within clusters still interfere.

Taken together, these results show that the shortage in bivalent formation observed in *axr1* mutants is not due to a general decrease in CO formation but rather to a mislocalisation of class I COs that tend to cluster together.

### Measurement of Recombination Rates in axr1 Mutants

The level of genetic recombination on several chromosomal intervals was measured using the Fluorescent-Tagged Lines (FTL) tool developed by Copenhaver et al. [50]. The FTL system is a visual assay based on segregation of genetically linked fluorescent proteins expressed in the pollen grains of the quartet mutant (*qrt1*), in which the pollen grains remain attached as tetrads. With these lines, a large number of meiotic products can be visually scored and then a subset of multiple CO events can be identified (two-, three-, and four-strand double COs in adjacent intervals and four-strand double COs within a single interval) ([50] and Table S3B). Six different intervals were used, either on chromosome 3 (I3b and I3c) or 5 (I5a, I5b, I5c, and I5d), with sizes ranging from 1,200 to 4,900 kb (Table S3A).

We first measured recombination rates for each interval using the standard Perkins genetic mapping equation [51]. As shown in Table 1, recombination rates in *axr1* vary differently depending on the interval tested, from 70% to 180% of the wild-type level. On average, *axr1* shows an increase in recombination, but these data should be taken with caution, as recombination measurements rely only on a subset of tetrads (the viable tetrads). Out of the six intervals considered, intervals located close to the telomeres (I3b and I5b) showed the most significant increase in recombination, whereas proximal intervals appeared less affected. This could indicate that the level of recombination is affected differently according to the location on the chromosomes, although additional data will be required to determine if telomere proximity increases CO frequency in the mutant.

**Table 1:** Recombination rates and interinterval interference.

Type	Intervals	*N*° of Tetrads^c^	d (CM)^d^	d ratio (*axr1*/wt)^e^	NPD Ratio^f^	*p* (NPDr = 1)^g^
Wt	I5a^a^	3,118	24.2	—	0.27	<10^−12^
	I5b^a^	3,118	15.5	—	0.37	<10^−3^
	I5a^b^	1,899	27.9	—	0.27	<10^−11^
	I5b^b^	1,899	16.7	—	0.27	<10^−3^
	I5d	8,947	8.6	—	0.21	10^−5^
	I5c	8,947	8.9	—	0.35	<10^−3^
	I3c	10,245	4.8	—	0.43	0.1
	I3b	10,245	17.0	—	0.30	<10^−18^
*axr1*	I5a^a^	1,263	17.0	0.7**	2.69	<10^−7^
	I5b^a^	1,263	23.7	1.5**	1.63	<10^−2^
	I5a^b^	1,107	24.4	0.9*	1.47	0.05
	I5b^b^	1,107	29.9	1.8**	1.18	0.05
	I5d	2,274	9.1	1.1	0.82	0.9
	I5c	2,274	8.8	1.0	0.74	0.7
	I3c	1,499	4.9	1.0	1.24	0.9
	I3b	1,499	20.9	1.2**	1.24	0.4

a, b Correspond to the data obtained for two independent experiments for intervals I5a and I5b.

c Only four-spore viable tetrads were considered. They correspond to 97% of the tetrads (*n* = 3,756) in wild type and 10% of the tetrads (*n* = 5,973) for *axr1*.

d Map distances were calculated using the Perkins genetic map equation [51] using raw data from Table S3B.

e Genetic distance ratio between *axr1* and wild type. It compares recombination rates between the two genotypes. Asterisks indicate significant differences between mutant and wild type (* *p*<0.05; ** *p*<0.01).

f Ratio between the observed number of double COs (based on NPD tetrad frequency) to the expected number of double COs under the hypothesis of no interference (see Table S5). The NPDr gives the strength of interference within the considered interval (no interference if the NPDr is equal to 1, absolute interference if the NPDr is equal to 0, negative interference if the NPDr is above 1).

g The *p* values indicate significant differences between IR and 1.

We then used the FTL data to estimate interference between COs occurring in adjacent intervals (Table 2 and Table S4). We calculated the Interference Ratio (IR) as defined by Malkova et al. [18], which compares the genetic length of one interval with and without the presence of a simultaneous event in the neighbouring interval. When the occurrence of a CO in one interval reduces the probability of a CO occurring in the adjacent interval, the IR is less than 1, indicating (positive) CO interference. When COs in the two adjacent intervals are independent of each other, the IR is 1, and if the presence of one CO in an interval increases the probability of an additional CO in the adjacent interval, the IR is greater than 1, indicating negative interference. As shown in Table 2, all wild-type IRs were less than 1, in agreement with the presence of CO interference. For *axr1*, however, all IRs increased dramatically and were statistically significantly different to wild type (*p*<0.0001, Table 2 and Table S4). In addition, all *axr1* IR values were greater than 1, although only one pair of intervals tested was significantly different from 1 (I5a I5b, first data set, IR = 1.63, *p* = 4×10^−3^). Therefore, in *axr1*, adjacent COs appear to occur more frequently than in wild type, which is in agreement with the previously observed clustering of class I COs scored cytologically (Figure 7). The cytologically observed clustering is occurring at a very small scale, namely a few hundred kb (on average 1,200 kb for HEI10 foci and 800 kb for MLH1 foci, see above), whereas in FTLs pairs of intervals correspond to more than 3,000 kb (I5cd, I3bc) and up to 7,500 kb (I5ab). Consequently, most of the clusters are expected to be present within a single interval and to only occasionally affect two adjacent intervals, which could explain why only one pair of intervals showed significant negative interference.

**Table 2:** Intra-interval interference.

Type	Adjacent Intervals	*N*° of Tetrads^c^	IR^d^	*p* (IR = IR wt)^e^	*p* (IR = 1)^f^
Wt	I5a/I5b^a^	3,118	0.47	—	<10^−8^
	I5a/I5b^b^	1,899	0.52	—	<10^−8^
	I5d/I5c	8,947	0.36	—	<10^−8^
	I3c/I3b	10,245	0.28	—	<10^−8^
*axr1*	I5a/I5b^a^	1,263	1.63	<10-4	4×10^−3^
	I5a/I5b^b^	1,107	1.25	<10-4	0.09
	I5d/I5c	2,274	1.26	<10-4	0.11
	I3c/I3b	1,499	1.35	<10-4	0.10

a, b Correspond to the data obtained for two independent experiments for intervals I5a and I5b.

c Only four-spore viable tetrads were considered. They correspond to 97% of the tetrads (*n* = 3,756) in wild type and 10% (*n* = 5,973) for *axr1*.

d The IR compares the genetic size of the first interval when a CO occurs in the adjacent interval to the genetic size of the same interval when no CO occurs in the adjacent interval (Table S4A).

e The *p* values indicate significant differences between *axr1* IR and wild-type IR for a given pair of intervals.

f The *p* values indicate significant differences between IR and 1.

Double CO events within a single interval can be detected using the FTLs if the two COs involve four different chromatids (Table S3B) because they will generate nonparental ditype (NPD) tetrads [50]. Interference within single intervals can be estimated by comparing the observed number of double COs (NPD frequency) to the expected number of double COs under the hypothesis of no interference [52]. The ratio between these two numbers (NPDr) gives the strength of interference within the considered interval, even if an important proportion of multiple COs will be silent. We calculated NPDr for all intervals considered for wild type and *axr1* (Table 1 and Table S5). In wild type, the NPDr indicated strong interference (NPDr close to 0.3) within all the intervals (except for I3c, which is too small for statistically meaningful data, Tables S3A and S5). In *axr1*, however, the NPDr increased systematically (between 0.7 and 1.47) and was mostly greater than 1. For two intervals (I5a and I5b), the NPDr values of 2.69 and 1.63 were statistically significant (*p*<0.01), showing negative interference (Table 2).

Thus, genetic analyses allowed us to measure negative interference in several of the intervals tested, confirming the CO clustering observed in cytology.

### Recombination Initiation Is Not Modified in axr1 Mutants

To verify whether the recombination defect in *axr1* could be linked to a defect in recombination initiation, we used two methods to investigate DSB formation. We first introgressed the *axr1* mutation into a *rad51* mutant, defective for meiotic DSB repair. In this mutant, DSBs are formed but are then repaired abnormally, leading to significant chromosomal defects (such as chromosome bridges and chromosome fragmentation) during anaphase I (Figure S5A). These chromosomal defects persisted in *axr1rad51*, showing that DSBs are present in the *axr1* mutant (Figure S5B). Second, we analysed the nuclear distribution of the DMC1 protein, a meiosis-specific recombinase that forms foci at recombination sites. The dynamics and number of AtDMC1 foci in *axr1* (237±40, *n* = 7) were indistinguishable from wild type (234±89, *n* = 28) (*t*, *p* = 0.9) (Figure S5). Thus, the meiotic defects observed in *axr1* are not correlated with a decrease in the amount of recombination initiation events.

### Synapsis Is Strongly Defective in axr1 But Chromosome Axes Are Normal

During meiotic prophase, chromosomes are structured in the context of a protein axis (the AE), which is crucial for most meiotic events, including meiotic recombination and synapsis [53],[54]. The meiotic chromosome axis is composed of specific AE proteins, such as ASY1 and cohesion proteins (REC8 and SCC3, [55],[56]). In wild-type meiotic cells, cohesins are loaded as early as premeiotic G1, whereas ASY1 appears at leptotene first as foci, then as a linear signal throughout the entire chromosome length (Figure S6A), in a pattern similar to that of cohesins (Figure S6C, [56]). As shown in Figure S6, the signal observed in *axr1* mutants cannot be differentiated from wild type, showing that no major alteration of the axis can be detected in *axr1* mutants.

**Figure 8:**
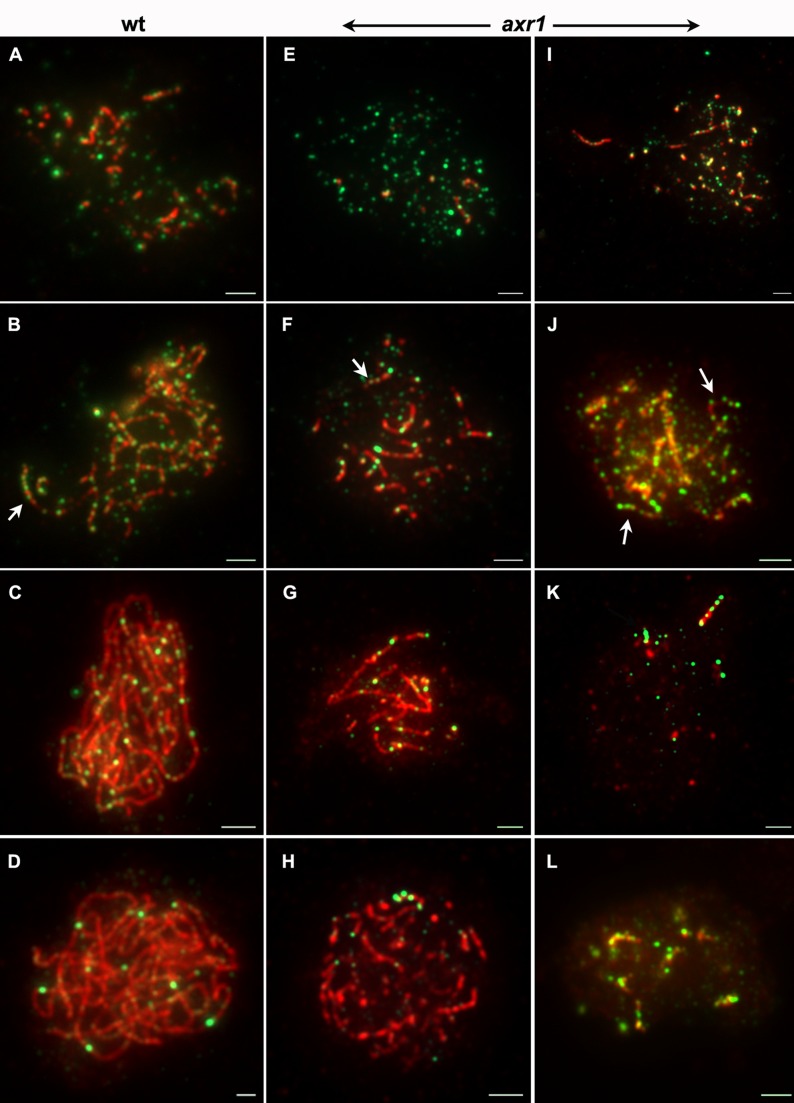
Synapsis is strongly perturbed in *axr1*, but HEI10 dynamics during early prophase are unchanged. ZYP1 and HEI10 proteins were co-immunolocalised on lipsol-spread chromosomes from wild-type (A–D) and *axr1* (N877989 allele, E–L) meiotic cells. The overlay of both signals is shown here (ZYP1 in red, HEI10 in green), but single channels can be found in Figures S7 and S8. In wild type as in *axr1*, ZYP1 appears on chromosomes as foci that quickly elongate, yielding a mixture of foci and short stretches (A, B, E, F, and I). Synapsis then progresses until complete synapsis is reached in wild type, defining the pachytene stage (C–D). In *axr1*, ZYP1 elongation can be detected, but full synapsis was never achieved (G, H, and K). In *axr1*, the ZYP1 signal is often uneven in thickness or forms dotted lines rather than a homogeneous and continuous signal (J and G). In addition, in some cases, only short and thick ZYP1 stretches were detected which could correspond to ZYP1 polycomplexes (L). During early zygotene, in wild type as in *axr1*, HEI10 forms numerous foci of variable sizes on chromatin (A, E, and I). Then, although synapsis progresses, combinations of large and small foci are observed, forming “strings of pearls” on ZYP1 stretches (B, F, and J, arrows). As meiosis progresses, a few bigger and brighter HEI10 foci can be observed in wild type (D) and in *axr1* (G, H, K, and L), which generally co-exist with smaller and fainter HEI1O foci (C, D, G, H, and K). Whereas this latter HEI10 pattern is associated with complete synapsis in wild type (C–D), synapsis is only partial in *axr1* (G, H, K, and L). Bar = 2 µm.

We then analysed the progression of synapsis by immunolocalisation of ZYP1, the *A. thaliana* CE component [57]. In wild type, ZYP1 appeared on chromosomes as foci that quickly elongated to yield a mixture of foci and short stretches of ZYP1 (Figure 8A,B, red signal and Figure S7). Synapsis then progressed until complete synapsis was reached, defining the pachytene stage (Figure 8C,D and Figure S7). In *axr1*, the early stages of synapsis could not be distinguished from wild type, showing a mix of foci and short ZYP1 stretches (Figure 8E,I and Figure S7). As meiosis progressed, ZYP1 elongation could be detected (Figure 8F–L and Figure S7), but full synapsis was never achieved (*n* = 66), confirming the synapsis defect detected after DAPI staining of meiocyte spreads (Figure 3). In addition, in approximately half of the cells, ZYP1 signals appeared strongly perturbed, uneven in thickness and forming dotted lines rather than a homogeneous continuous signal (Figure 8J or G and Figure S7). In some cases, only short and thick ZYP1 stretches were detected. These could correspond to ZYP1 poly-complexes rather than to CE polymerisation (Figure 8L and Figure S7).

### CO maturation and Synapsis Can Be Uncoupled in *axr1*

To follow the progression of meiotic recombination events, we co-immunolocalised ZYP1 and HEI10, using a lipsol spreading protocol that has the advantage of allowing the simultaneous detection of these two proteins [58] but also the disadvantage of preventing examination of prophase after pachytene [59]. As mentioned above, HEI10 is detected as foci on meiotic chromosomes from leptotene to diakinesis, and its dynamics reflect the progression from early recombination intermediates to mature class I COs [48]. During leptotene and early zygotene, HEI10 forms numerous foci of variable size on chromatin (Figure 8A and Figure S8). Then, during synapsis initiation, bigger and brighter HEI10 foci appear, often co-localising with synapsed regions (Figure 8B and Figure S8). At this stage and later on, a combination of large and small foci are observed, forming “strings of HEI10 pearls” on ZYP1 stretches (Figure 8B,C and Figure S8B,C, arrows). At late pachytene, only a few bright HEI10 foci, corresponding to mature class I COs, are retained (Figure 8D and Figure S8). Nevertheless, during most of the pachytene stage, bright HEI10 foci are present, together with faint HEI10 signal marking the CE (Figure S8C,D). In *axr1*, the dynamics of HEI10 progression were the same as in wild type with HEI10 detected as multiple foci during early prophase stages (Figure 8E,I and Figure S8). Brighter foci then appeared as synapsis progressed, also forming a string of pearls on ZYP1 stretches (Figure 8J and Figure S8, arrows). A subset of very bright foci was retained at the later stages (Figure 8G,H,K,L and Figure S8). We noticed that at these late stages (based on the HEI10 pattern), the level of synapsis varied considerably from one cell to another. In addition, although these late HEI10 foci were always observed on ZYP1 stretches, the reverse was not true and ZYP1 stretches without late HEI10 signals were observed (see, for example, Figure 8H, where four late HEI10 foci are clustered on a single ZYP1 stretch, whereas many ZYP1 stretches are deprived of HEI10 foci). Therefore, it appears that class I CO clustering in *axr1* is correlated with strong synapsis defects, but cannot be explained by the limited extension of the SC.

### Meiotic Defects in the *axr1* Mutant Are Epistatic to Those of a Cullin 4 Mutant

Because neddylation is known to regulate the activity of CRLs, we investigated whether *axr1* meiotic defects are dependent on a specific CRL. In *A. thaliana* only four cullins are neddylated: cullin 1, cullin 3A, cullin 3B, and cullin 4 [33]. To identify possible AXR1 downstream players, we scored cullin-deficient lines for meiotic defects. Complete suppression of any of cullin functions (null *cul1* or *cul4* or the double *cul3a cul3b* mutants) is lethal, but various genetic backgrounds deficient in cullin activities are available

We first investigated meiosis of the auxin response defective *cul1* mutant alleles—*cul1–6* [60], *axr6-2/N3818* [61], and *axr6-3/eta1* [62]—and observed perfectly normal meiosis (not shown). Next, considering cullin 3 activity, we analysed the CUL3a/3b hypomorphic mutant [*cul3w* (*cul3a3cul3b1*)] described for its defects in various aspects of the ethylene biosynthesis pathway and root development [63]. *cul3w* plants also showed normal meiotic development of male meiocytes (not shown).

**Figure 9:**
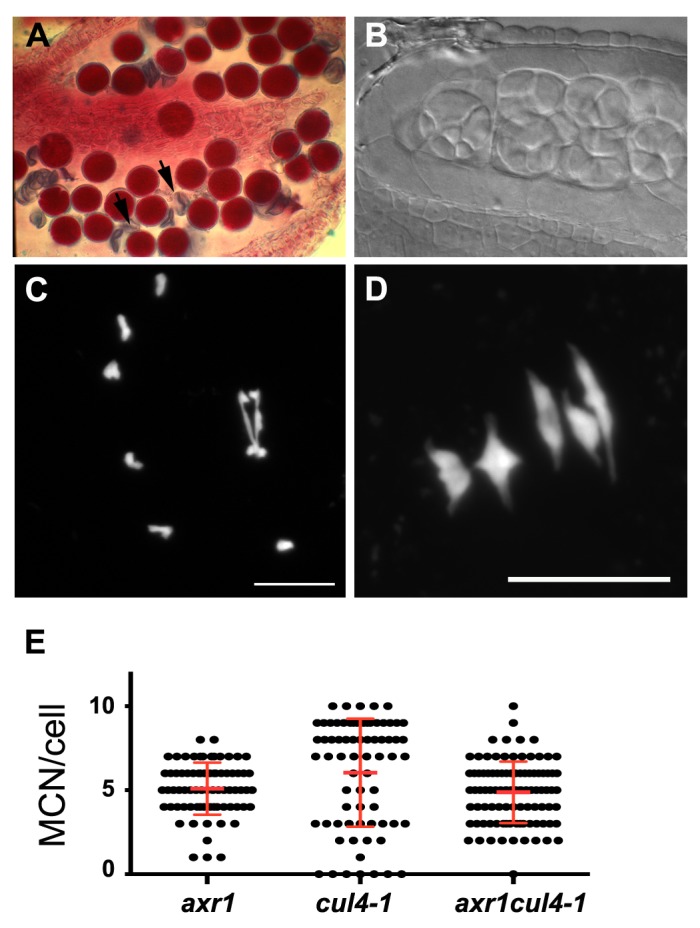
CULLIN4 is involved in meiotic recombination in the same pathway as *axr1*. In the *cul4-1* mutant, a mixture of viable (purple) and dead (arrow) pollen grains can be seen in the anthers after Alexander staining (A). This is correlated with the production of aberrant tetrads and polyads of microspores (B). DAPI staining of the meiotic chromosomes revealed a defect at metaphase I (C) in bivalent formation, which is quantified in (E). Bar  =  10 µM.

Finally, we analysed the *cul4-1* mutant in which a T-DNA is inserted occurred in the 12th exon of the gene, leading to aberrant *CUL4* mRNA expression, which varies depending on the developmental stage [64]. We observed significant male and female gametophyte abortion in *cul4-1* (shown for the male, compare Figure 9A to Figure 2C). Although in wild type only balanced tetrads of microspores were observed, asymmetric tetrads and polyads were seen in *cul4-1* mutants (compare Figure 9B to Figure 2E). Male meiosis was then investigated. The first stages of meiosis proceeded normally in *cul4-1* mutants, however we observed metaphase I phenotypes reminiscent of the *axr1* defects, with a large proportion of cells showing a clear reduction in bivalent formation (Figure 9C). The MCN per meiotic cell in *cul4-1* (6±3.2, *n* = 71) was significantly different from wild type (8.9±0.9, *n* = 51, *p*<0.0001), and slightly different from *axr1* (5.1±1.5, *n* = 74, *p* = 0.02). Nevertheless, the number of MCN per cell in *cul4-1* was far more variable than in *axr1* (Figure 9E), due to an overrepresentation of cells with wild-type levels of chiasmata (Figure 9D,E). We then introgressed the *axr1* mutation (N877898) into *cul4-1* and found that the double mutant cannot be distinguished from the single *axr1* in terms of meiotic phenotype (not shown), the average level of MCN per cell (4.9±1.8, *n* = 98, *p* = 0.412), and in terms of variability of the values (Figure 9E), showing that *axr1* is epistatic to *cul4-1*. Overall, our results suggest that AXR1 acts during meiotic recombination through the activation of a CRL4 complex.

## Discussion

### AXR1 Controls the Localisation of Class I COs During Meiosis

We observed that in *axr1* mutants, meiotic nondisjunction is correlated with defects in bivalent formation. However, our results indicate that the general level of meiotic recombination in *axr1* is close to that of wild type, as we showed that COs are mostly under the control of the ZMM pathway and that their average number, revealed by MLH1 and HEI10 foci, is unchanged. Furthermore, these CO events show a completely aberrant distribution in *axr1*. First, cytogenetic data showed that clustered MLH1 or HEI10 foci are observed in approximately 50% of the meiocytes (Figure 7B). Second, genetic data showed that adjacent COs in most tested intervals no longer display genetic interference, showing that CO distribution is abnormal in *axr1*. More strikingly, in several intervals, strong significant negative interference was detected, a genetic demonstration of CO clustering. We can therefore conclude that COs in *axr1* tend to cluster together and that the observed shortage in bivalent formation is not due to a global decrease in meiotic recombination but rather mislocalisation of these events, resulting in a loss of the obligatory CO.

Very little information is available on the mechanisms that control CO distribution during meiosis. Nevertheless, it has been known for a long time that COs are not randomly distributed among chromosomes, as in most organisms, adjacent COs display interference and therefore tend to be evenly spaced within chromosomes [13]. In addition, the phenomenon of the “obligatory CO” (or CO assurance) ensures the formation of at least one CO per bivalent, whatever the total number of CO precursors per cell. The relationship between these two phenomena is still under debate [23], but recent modelling analyses suggested that the obligatory CO is a direct consequence of interference [65]. Numerous mutants with altered interference were described, but they nearly always also change CO rates, either because of increased MUS81-dependant COs [66]–[68] or because they are defective in the ZMM CO pathway (see, for example, [48],[69]). One possible exception is the *Saccharomyces cerevisiae pch2* mutant, for which two independent studies showed that CO interference is alleviated without changes to meiotic recombination rates, at least on the smallest yeast chromosome (III) [70],[71]. Nevertheless, the generalisation of this observation to the whole genome seems unlikely [71].

To our knowledge, *axr1* is therefore the first mutant that specifically modifies the localisation of class I COs, changing interference among them and resulting in the loss of the obligatory CO, but without changing the global average number of CO events. Thus, AXR1 is a key regulator of meiotic recombination outcomes.

From our data, we can exclude that the meiotic defects observed in *axr1* are due to a major decrease in DSB formation or to a drastic mislocalisation of these events (Figure S5). We have also shown that *axr1* meiotic defects are not associated with major chromosome axis defects (Figure S6), but instead with major perturbations in the polymerisation of the SC CE (Figure 8).

The relationship between CO control and SC polymerisation is a long-standing question in the field of meiosis [72]. In yeast, SC polymerisation is not necessary for CO interference, as it occurs after CO patterns have been imposed [73],[74]. In *Caenorhabditis elegans*, however, it was recently shown that the SC central region limits the formation of COs and imposes total interference [75]. This could also be the case in rice, where *zep1* mutants (ZEP1 being the rice CE *ZIP1* homologue) show an increase in chiasma formation at diakinesis [76]. This suggests that, in plants as in *C. elegans*, SC polymerisation could be necessary to limit CO formation. However, in *Arabidopsis*, ZYP1 appears to be required to prevent non-HR rather than acting on homologous CO formation [57]. To further complicate our understanding of the relationship between polymerisation of the SC CE and CO controls, in yeast, SC polymerisation requires the stabilisation of recombination intermediates by the ZMM proteins [77],[78]. In *Arabidopsis*, SC polymerisation is also dependent on the formation of HR intermediates, as no synapsis is observed either in *spo11*, *dmc1*, or *rad51* mutants where recombination is either not initiated or is blocked at the invasion step. However, *Arabidopsis zmm* mutants all display normal synapsis [20],[48],[69], showing that SC polymerisation in these species depends on the formation of recombination intermediates, but not on their stabilisation by the ZMMs.

The limited synapsis progression observed in *axr1* mutants therefore suggests that recombination only proceeds far enough in a limited fraction of the genome where SC can polymerise and COs are formed. This appears to explain what is seen in Figure 8K, where mature HEI10 foci are concentrated on a few ZYP1 stretches. In that sense, aberrant SC formation illustrates that either recombination is blocked in a portion of the genome or that only a limited portion of the genome is competent to support recombination maturation, resulting in the loss of the obligatory CO. Nevertheless, the observation of nuclei where mature HEI10 foci are clustered on a single ZYP1 stretch, whereas the level of synapsis is high (as illustrated on Figure 8H) shows that the amount of ZYP1 polymerisation can be uncoupled from clustering of mature HEI10 foci. This suggests that CO clustering in *axr1* is not only a consequence of limited synapsis progression.

It is interesting to note that, within clusters of more than two class I CO foci, the distance among foci is not random, showing that they still display interference. During wild-type meiosis, no more than two adjacent foci could be scored, showing that these events are either less frequent or much more distant than in *axr1*. In this latter case, our results suggest that interference strength is considerably modified in *axr1*, resulting in CO clustering, at least in some areas of the genome. In *Arabidopsis*, interference strength is not uniform within chromosomes and increases toward the chromosome extremities [79]. This suggests that regional modification of interference parameters could be affected in *axr1*. In the future, it will therefore be crucial to determine whether CO clustering in *axr1* is region-specific or not. Regardless, the average number of final CO events is unchanged in *axr1*, suggesting that (i) the total number of class I COs is precisely controlled, (ii) this control is still active in *axr1*, and (iii) the mechanism underlying this CO homeostasis is independent of the obligatory CO mechanism.

### AXR1 Acts on Meiotic Recombination Through Activation of a CRL4 Complex

Neddylation stimulates several subclasses of cullin RING Ub ligases. We provide evidence that during meiotic recombination neddylation acts through cullin 4 activation to regulate the localisation of class I COs. Cullin 4 is a widely conserved cullin, involved in a large range of cellular and developmental controls, many of which are associated with genome integrity maintenance [80].

CRL4 complexes are composed of a CUL4 scaffold, a small RING domain containing RBX1 protein, a WD40-like repeat-containing adaptor DDB1 (DNA-damage binding 1), and a substrate receptor subunit called DWD (DDB1-binding WD40 protein) or DCAF (DDB1- and CUL4-associated factor) [80]. Evidence for CRL4 functions in genome integrity control come from multiple sources and concern mostly cell responses to UV damage and replication controls by regulating the accumulation of the replication licensing factor CDT1. For example, DDB1- and/or CUL4A-depleted human cells accumulate DSBs and have an activated ATM-ATR cell cycle checkpoint [81]. The budding yeast *cul8* mutants (cullin 8 is thought to be the functional homologue of cullin 4 in *S. cerevisae*) also accumulate DNA damage [82]. In fission yeast, mutation in *Ddb1* increases the spontaneous mutation rate by more than 20-fold and prevents premeiotic S phase entry [83]. CRL4 activity is also required for the NER pathway by controlling the detection and processing of DNA lesions induced by UV in plants [41],[84]–[86], but also in mammals, as loss of *CUL4A* in mice leads to an increase in susceptibility to UV skin cancer [87]. Evidence for the role of CRL4 complexes in DSB repair was also provided in *Drosophila*, where DDB1 depletion promotes loss of heterozygosity in somatic cells [88]. In addition, CRL4s complexes may also be involved in HR regulation, as in fission yeast, *ddb1* mutants are defective in HR probably by regulating the pool of available dNTPs [89].

Interestingly, we observed that hardly any COs are retained in double *axr1zmm* mutants, suggesting that the MUS81 recombination pathway may be shut down in *axr1*. Because this pathway accounts for only a small proportion of all COs in *Arabidopsis* [14],[22], this disruption would not have a strong impact on meiosis. However, it could have a dramatic effect on somatic DNA repair, as the MUS81 pathway is one of the major pathways of somatic HR in eukaryotes [90]. It would therefore be interesting to study the involvement of AXR1 in somatic DNA recombination, above all considering that Dohmann and collaborators observed DSB accumulation in *axr1* (*axr1-3* and *axr1-12*) somatic cells [91].

Considering the crucial role of CRL4s in genome maintenance and the activation of DNA repair pathways including HR, it is hardly surprising to find that it is involved in the regulation of meiotic HR in *Arabidopsis*. Considering the conservation of CRL4 functions across kingdoms, it is likely that the regulation of meiotic recombination by one (or several) CRL4 complex(es) will be also observed in other eukaryotes. Indeed, two converging studies in mice recently showed that cullin 4 is also required for meiosis also in mammals, as depletion of *Cul4a* (one of the two mammalian *Cul4* genes) led to male infertility [42],[43]. Whether this infertility is associated with early recombination [42] or later CO resolution defects [43] is still under debate. Nevertheless, the observation that MLH1 foci number is unchanged in *cul4a* but that a fraction of meiotic cells show pachytene bivalents without any MLH1 foci [43] is reminiscent of our data on *axr1.* Therefore, we propose that neddylation is acting on one or several CRL4 complex(es) to regulate the localisation of class I COs not only in *Arabidopsis* but also in mammals. In *A. thaliana*, there are more than 85 substrate receptor DWD domain proteins that can assemble with DDB1A or DDB1B or directly with CUL4-RBX1 to form CRL4 complexes [92],[93]. Further studies will be necessary to identify which of these is acting during meiosis.

## Materials and Methods

### Plant Material

Ws-4 lines (including EGS344, EIC174, and EVM8) were obtained from the Versailles collection of *Arabidopsis* T-DNA transformants available at http://www-ijpb.versailles.inra.fr/en/sgap/equipes/variabilite/crg/ [94].

Col-0 lines [including N877898 (Sail_904E06) and N3076 = *axr1-12*] were obtained from the collection of T-DNA mutants from the Salk Institute Genomic Analysis Laboratory (Columbia accession) (SIGnAL, http://signal.salk.edu/cgi-bin/tdnaexpress) [95] and provided by NASC (http://nasc.nott.ac.uk/).

Other mutant alleles used in this study are as follows: *msh4^Ws^* (EXY25) [48], *msh5^Col^* (SALK_026553) [96]; *hei10^Ws^* (EQO124) [48], *zip4^Col^* (SALK_068052) [69]; *mer3^Col^* (*mer3-2*, SALK_091560) [20], *mlh1^Col^* (SK_25975) [48], *mus81^Col^* (SALK_107515) [14], *rad51^Col^* (Gabi_134A01) [97], *mre11^Col^* (*mre11-4*, Salk_067823), *cul1-6^ Col^* [60], *axr6-2^ Col^* (N3818) [61], *axr6-3^ Col^* (*eta1*) [62], *cul3w ^Col^* [63], and *cul4-1 ^Col^* [64].

### Growth Conditions

Plants were grown in a greenhouse (photoperiod 16 h/d and 8 h/night; temperature 20°C day and night; humidity 70%).

### *AXR1* Cloning

Screening for *A. thaliana* T-DNA (*A. tumefaciens* transferred DNA) insertions that provoke meiotic defects, we isolated three mutant lines: EGS344, EIC174, and EVM8. They all segregated 3∶1 for reduced fertility, meiotic defects, and a bushy vegetative phenotype. Linkage analysis (as described by Grelon et al. [98]) showed that none of the mutations were linked with a T-DNA insertion. We therefore undertook a rough positional cloning of the three mutations as described by De Muyt et al. [99]. The most closely linked marker was chr1_02991901 for all three mutants (based on 31 F2 mutant plants for EVM8, 31 for EGS344, and 31 for EIC174). Fine gene mapping was then carried out as described by De Muyt et al. [99] using chromosome 1 microsatellite markers located between 1,243,352 and 1,573,000 bp.

Among the predicted genes by TAIR10 SeqViewer server (http://www.arabidopsis.org/), we retained *AXR1* (At1G05180) as the best candidate, as *axr1* mutants were previously shown to display the same vegetative developmental defects as EGS344, EIC174, and EVM8 [44],[46]. Sequencing of At1g05180 in the three mutant lines showed that all three are disrupted in this open reading frame (see below).

We further analysed the *axr1* reference allele (*axr1-12*) and another insertion line (Sail_904E06) available in the public databases (http://signal.salk.edu/). They all displayed the same meiotic phenotype as the previously isolated lines.

### Molecular Characterisation of *axr1* Alleles

Sequencing of At1g05180 in the EIC174 mutant line revealed a single nucleotide insertion in exon 6 (position 1364 of the genomic sequence, corresponding to nt 688 in the cDNA), leading to a premature stop codon (a 222 aa protein is produced instead of 540 aa in wild type). In the EGS344 mutant, a deletion of 898 bp (from nucleotide 91 of the genomic sequence) together with an insertion of *Agrobacterium* plasmid Ti DNA disrupts At1g05180 (Figure S1). In the EVM8 line, an in-frame deletion of 312 bp occurred between exons 3 and 4, generating a 20 aa deleted protein. Details are shown in Figure S1.

In *axr1-12*, corresponding to the N3076 line, a single C-T nucleotide substitution in position 1295 of the cDNA occurred, leading to a premature stop codon (415 aa instead of 540), as described by Leyser et al. [44]. In N877898, corresponding to the Sail_904E06 line, a T-DNA insertion occurred in intron 11.

Sequence references are as follows: Tair Accession 4010763662 for the genomic sequence, and Tair Accession 4010730885 for the cDNA sequence.

### PCR Genotyping of Mutant Lines

For EGS344 and EVM8, wild-type alleles were amplified with primers 05180-P1 (ACCCTGATTGAAGAAAAGTCT) and 05180-P2 (CGGAGGTCGTCAAGAAAA) (60°C, 30 PCR cycles, 1,200 bp). The EGS344 mutant allele was amplified with primers 05180-P1 and 05180-AgroP1 (ACATCACAGCACCTCGATCCTGG) (60°C, 30 PCR cycles, 300 bp). The EVM8 mutant allele was amplified with 05180-P1 and 05180-P2 (60°C, 30 PCR cycles, 980 bp)

For N877898, the wild-type allele was amplified with primers N877898U and N877898L (60°C, 30 PCR cycles, 957 bp). The mutant allele was amplified with primers N877898L and Lb3SAIL (TAGCATCTGAATTTCATAACCAATCTCGATACAC) (60°C, 30 PCR cycles, 500 bp).

For all other genotypes, the primer list and PCR amplification conditions are shown in Table S6.

### Genetic Analyses

#### Recombination and interference measurements

The six intervals used in this study correspond to intervals I5a, I5b, I5c, I5d I3b, and I3c, described by Berchowitz et al. [50] and in Table S3A.

We produced plants *qrt^−/−^* N877898^+/*−*^, and *qrt^−/−^* N877898^+/*−*^ RYC/RYC. We crossed these two plants and in the progeny analysed tetrad fluorescence of semi-sterile plants *qrt^−/−^* N877898*^−^* ^/*−*^ RYC/+++ or fertile plants either *qrt^−/−^* N877898^+/*−*^ RYC/+++ or *qrt^−/−^* N877898^+/+^ RYC/+++. Plants were grown in a greenhouse. Tetrad analyses were carried out as described in [50]. The resulting tetrad data (Table S3B) were analysed as described by Berchowitz et al. [50]. In brief, map distances were calculated using the Perkins mapping equation based on the measurement of the frequency of tetratype (T), parental (P), and NPD combinations of markers [d(cM) = (100 [6NPD+T])/(2[P+NPD+T])] [51]. Interference was then measured by comparing CO frequency in an interval when the adjacent interval had no CO to the CO frequency when the adjacent interval does have a CO, as described by Malkova et al. [18]. We calculated the ratio of these genetic distances and statistically compared these ratios as described by Berchowitz et al. [50] and using Stahl Lab Online tools (http://www.molbio.uoregon.edu/~fstahl/). Another estimate of interinterval interference via the coefficient of coincidence is shown in Table S4B. In addition, we estimated the level of intra-interval interference by calculating the NPD ratios (Table S5), which compares the number of observed double COs within a single interval (NPD) to the expected number of double COs under the hypothesis of no interference [52],[100].

#### Interference analysis of *axr1* using the HEI10 and MLH1 foci patterns

Given a cluster of at least three MLH1 or HEI10 foci, we asked whether the internal foci were distributed as happens in the absence of interference. Because these clusters were small, under the hypothesis *H_0_* of no interference, the foci should be distributed uniformly. Considering first the clusters formed with three foci, let *d_1_* and *d_2_* be the two distances between adjacent foci. After normalization, we have *d_1_*+*d_2_* = 1. Then, we introduced the statistic *S* that corresponds to the sum of the squared centred deviations, normalized by the variance under *H_0_*. Because the mean (respectively, the variance) of a uniformly distributed random variable in [0;1] is 0.5 (respectively, 1/12), *S* = [(*d_1_*–0.5)^2^+(*d_2_*–0.5)^2^] * 12. This statistic can be generalized to clusters with *k*+2 foci (*k*+1 distances, *d*_1_+*d*_2_+…+*d_k_*_+1_ = 1): *S* = [(*d_1_*–1/(*k*+1))^2^+(*d_2_*–1/(*k*+1))^2^+… +(*d_k_*_+1_–1/(*k*+1))^2^] * (*k*+1)^2^ * (*k*+2)/*k*.

The normalization is chosen so that under *H_0_* each distance contributes to the same average to the statistic, regardless of the value of *k*. The total statistic *S* for the test is simply obtained by summing over all clusters. It is a random variable whose distribution we obtained by direct simulation under *H_0_* of 10^5^ datasets having the same values of *k* as in the experimental measurements. Small experimental values of *S* correspond to foci more regularly distributed than expected. The *p* value for our test is given by the proportion of simulated statistics smaller than that of the experimental data.

### Antibodies

The anti-ASY1 polyclonal antibody was described by Armstrong et al. [101]. It was used at a dilution of 1∶500. The anti-ZYP1 polyclonal antibody was described by Higgins et al. [57]. It was used at a dilution of 1∶500. The anti-DMC1, anti-MLH1, and anti-HEI10 antibodies were described by Chelysheva et al. in [69], [49], and [48], respectively. These were used at a dilution of 1∶20, 1∶200, and 1∶200, respectively. The anti-REC8 polyclonal antibody was described by Cromer et al. [102] and the anti-SCC3 by Chelysheva et al. [56]. These were used at a dilution of 1∶250 and 1∶500, respectively.

### Microscopy

Comparison of the early stages of microsporogenesis and the development of PMCs was carried out as described in Grelon et al. [98]. Preparation of prophase stage spreads for immunocytology was performed using Carnoy's fixative and acetic acid chromosome spreads [59], except for DMC1 detection and double HEI10/ZYP1 immunolabelling where lipsol spreading and paraformaldehyde fixation were used [58]. Chiasma numbers were assessed by analysing metaphase I spread PMC chromosomes stained with DAPI, as described by Sanchez-Moran et al. [103]. In brief, a rod bivalent stands for a single chiasma, whereas a ring bivalent as two (one on each arm).

Observations were made as described by Chelysheva et al. [48].

## Supporting Information

Figure S1 Molecular characterisation of the *axr1* alleles. In EIC174, a single nucleotide (A) insertion occurred in exon 6 (position 1364 of the genomic sequence, in red), leading to a premature stop codon (a 222 aa protein is produced instead of 540 aa in wild type). In EVM8, a large in-frame deletion of 312 bp (in blue) generates a 20 aa truncated protein. In EGS344, a 907 bp deletion (in green) associated with an *Agrobacterium* Ti plasmid DNA insertion (*) occurred in the 5′ end of the gene.
(DOCX)

Figure S2 Phenotype of *axr1* mature plants. (A) *axr1* mutants display strong vegetative defects. All plants shown are 6 wk old. Upper panel, *axr1* allelic series. Lower panel, *axr1* mutants compared to their respective wild-type strain (Col-0 or Ws-4). (B) *axr1* mutants produce less seeds than wild type.
(TIF)

Figure S3 MLH1 foci localise on chiasma-containing arms in wild type and *axr1*. MLH1 was immunolocalised on acetic acid spread chromosomes from wild type (wt, Col-0) and *axr1* (N877898) at diakinesis. Because adjacent univalents cannot be distinguished from bivalents, we selected only the MLH1-labelled bivalent-like structures (arrows) and scored where MLH1 foci occurred. In both genotypes, 100% of the MLH1 foci (*n* = 246 for wild type, *n* = 44 for *axr1*) were detected on connected chromosome arms and never on a free chromosome.
(TIF)

Figure S4 Class I CO cluster characterisation. (A) The distance between two adjacent foci does not vary significantly with the cluster type. The distance between two adjacent foci (measured in µm and divided by the total size of the chromosome axis) was measured in clusters containing two, three, or four clustered foci. The *x* axis is the total number of foci in the considered clusters. All measures were undertaken in the N877898 allele. (B) Class I CO cluster lengths increase proportionally with the foci number. In the graph below, the mean size of the HEI10 or MLH1 clusters (calculated in µm and divided by the total size of the chromosome axis) is given according to the number of foci present in the cluster. All measures were undertaken in the N877898 allele.
(TIF)

Figure S5 Early recombination events are not altered in *axr1*. (A and B) DAPI staining of anaphase I meiocytes from *rad51* (A) and *axr1rad51* (B) mutants. *rad51* is defective in meiotic DSB repair as shown by the major chromosomal defects observed at meiosis (fragmentation, A). Introgression of the *axr1* mutation (N877898 allele) does not rescue these defects (B), showing that meiotic DSB are present in *axr1*. Bars = 10 µm. (C–F) Lipsol chromosome spreads of *Arabidopsis* meiocytes stained with DAPI (C and E) and immunolabelled with the anti-DMC1 antibody (D and F). *axr1* meiocytes (N877898 allele, E and F) show wild-type–like DMC1 staining. Bar = 5 µm.
(TIF)

Figure S6 No major axis defect can be detected in *axr1*. Immunolocalisation of ASY1 (A and B), REC8 (C and D), and SCC3 (E and F) in wild type (A, C, and E) and *axr1* (B, D, and F) prophase meiotic cells (N877898 allele). Bar = 5 µm.
(TIF)

Figure S7 Synapsis is strongly perturbed in *axr1*. ZYP1 was immunolocalised on lipsol spread chromosomes from wild-type (A–D) and *axr1* (N877989 allele, E–L) meiotic cells. This figure corresponds to the red channel from Figure 8.
(TIF)

Figure S8 HEI10 dynamics during early prophase is unchanged in *axr1*. HEI10 was immunolocalised on lipsol spread chromosomes from wild-type (A–D) and *axr1* (N877989 allele, E–L) meiotic cells. This figure corresponds to the green channel from Figure 8.
(TIF)

Table S1 Average MCN and average bivalent number per meiocyte.
(DOCX)

Table S2 Interfoci distance within class I CO clusters. In *axr1* (N877898 allele), HEI10 and MLH1 foci form clusters in approximately half of the pachytene/diplotene meiocytes. To estimate interference between adjacent foci within the clusters, we measured the distance between two adjacent foci in clusters containing more than two foci.
(DOCX)

Table S3 Recombination dataset. (A) The localisation of the six FTL intervals used in this study are shown below on the five *Arabidopsis* chromosomes. They correspond to three pairs of linked intervals—I5a I5b, I5c I5d, and I3b I3c—described by Berchowitz and Copenhaver [50]. The position (in bp) of each transgene encoding red, yellow, or cyan fluorescent proteins (filled circles) is shown. The size of each interval is given in the accompanying table. The map was obtained using the Chromosome Map Tool from http://www.arabidopsis.org/jsp/ChromosomeMap/tool.jsp. (B) To measure recombination rates, we produced plants homozygous for the *quartet* (*qrt*) mutation, heterozygous for *axr1* (N877898 allele), and carrying or not three linked fluorescent markers (R, red; Y, yellow; C, cyan): *qrt* ^−/−^ *axr1* ^+/−^ and *qrt* ^−/−^ *axr1* ^+/−^ RYC/RYC. The *qrt* mutation allows the four pollen grains from a single meiosis to be maintained together. We crossed these two plants and in the progeny analysed tetrad fluorescence of mutant plants (*qrt* ^−/−^ *axr1* ^−/−^ RYC/+++) or fertile plants (wt) (either *qrt* ^−/−^ *axr1* ^+/−^ RYC/+++ or *qrt* ^−/−^ *axr1* ^+/+^ RYC/+++). Plants were grown in a greenhouse, and tetrad analyses were carried out as described by Berchowitz and colleagues [50]. The distribution of the markers within the tetrads and the resulting distribution of colours vary depending on the number, localisation, and chromatids involved in recombination. The different possibilities are indicated by drawings and the number of tetrads in each phenotypic class is shown in the tables below. Two replicates were made for the I5a I5b intervals.
(DOCX)

Tables S4 Interinterval interference analyses. Genetic interference among COs occurring in two linked intervals was calculated using two methods: (A) The IR. The IR [18] compares the genetic size of an interval (d(Ia), in cM calculated using the Perkins equation [51]) when a CO occurs in an adjacent interval (d(Ia) with CO in Ib) to the genetic size of the same interval when no CO occurs in the adjacent interval (d(Ia) without CO in Ib). The ratio of these two distances, called the IR, gives a measurement of the strength of interference between the two intervals. When there is no interference, the ratio is equal to 1, whereas the ratio is below 1 when there is interference. Ratios above 1 indicate negative interference, indicative of more adjacent C0s than expected. We compared each IR to 1 [*P*(IR = 1)] and compared mutant IR to wild-type IR [*P*(IR = IR wt)]. Calculations and statistical analyses were performed according to Berchowitz and Copenhaver [50] using Stahl Lab Online tools (http://www.molbio.uoregon.edu/~fstahl/). In wild type, IRs were statistically below 1 for all pairs of intervals considered, indicative of interference among COs. In *axr1*, IRs increased systematically and were always greater than 1, but the difference with 1 was significant (*p*<0.01) only for intervals I5a I5b and only for one replicate of the experiment. (B) The coefficient of coincidence. The coefficient of coincidence (c.o.c.) compares the observed frequency of double COs in the two adjacent intervals [f(Ia and Ib) observed] to the expected frequency of double COs if there were no interference [f(Ia and Ib) expected]. This last frequency is the product of the frequency of COs in each single interval [f(Ia) and f(Ib)]. The c.o.c. corresponds to (f observed/f expected). When interference is absent, the c.o.c. is equal to 1. When interference is total, the c.o.c. is equal to 0. For wild type, this index varied from 0.37 to 0.63, indicative of interference. In *axr1* the index was always above 1, which shows that double COs in adjacent intervals are more frequent than expected.
(DOCX)

Table S5 Intra-interval interference analyses. Interference was measured within a single interval, by comparing the observed number of double COs [based on NPD frequency (NPD observed) to the expected number of double COs under the hypothesis of no interference (NPD expected)] [52]. The ratio between these two figures (NPDr) gives the strength of interference within the considered interval. The NPD tetrads correspond to h, i, j, k, and l classes from Table S2. In wild type, NPDr indicate strong interference (NPDr close to 0.3) within all the intervals (except for I3c, which is too small to give statistically meaningful figures). In *axr1*, NPDrs increased systematically (between 0.7 and 2.69) and were generally greater than 1, indicative of a trend toward negative interference (more double COs in a single interval than expected). However, statistical analyses on NPDr (Stahl Lab Online tools, http://www.molbio.uoregon.edu/~fstahl/) showed that these values are statistically different from 1 (*p*<0.01) only on I5a and I5b (one of the two replicates).
(DOCX)

Table S6 Primer sequences and PCR conditions for mutant genotyping.
(DOCX)

## Abbreviations

CO: crossover
CRL: Cullin RING ligase
D-loop: displacement loop
DSB: double-strand break
HR: homologous recombination
IR: interference ratio
MCN: minimum chiasma number
NCO: gene conversion not associated with CO
NPD: nonparental ditype
PD: parental ditype
SDSA: synthesis-dependent strand annealing
ssDNA: single stranded DNA
T: tetratype
