# Supplementary material for: Crossover Localisation Is Regulated by the Neddylation Posttranslational Regulatory Pathway
Source: PLoS Biol. 2014 Aug 12;12(8):e1001930. doi: 10.1371/journal.pbio.1001930 (PMC4130666; doi:10.1371/journal.pbio.1001930)
Supplement: Figure S1 — Molecular characterisation of the axr1 alleles. In EIC174, a single nucleotide (A) insertion occurred in exon 6 (position 1364 of the genomic sequence, in red), leading to a premature stop codon (a 222 aa protein is produced instead of 540 aa in wild type). In EVM8, a large in-frame deletion of 312 bp (in blue) generates a 20 aa truncated protein. In EGS344, a 907 bp deletion (in green) associated with an Agrobacterium Ti plasmid DNA insertion (*) occurred in the 5′ end of the gene. (DOCX) [file pbio.1001930.s001.docx]

**Figure S1: Molecular characterisation of the *axr1* alleles**

| **Sequence: AT1G05180.1** | | |
| --- | --- | --- |
| **Name** |  | AT1G05180.1 |
| **Tair Accession** |  | Sequence:4010763662 |
|  |  |  |

  1  TTTATCAGTT CACCGGAGGC AAAAATCGTC TCTCGCTTGA GCTGCGAAGA
  51 TGCAAGCAGT AAAAAGATCC AGGAGACATG TTGAAGAAGA G*CCAACAATG
 101 GTAGAACCTA AAACCAAGTA CGATCGTCAG CTCAGGTATA CATACTCTTT
 151 TTCCTTAAAC TCTACTTCGA ACTACTCTTG TCGGGAGGAA AATTGATGAA
 201 GTAGTAATCG TTGGGTTGAC TGATTAGGAT TTGGGGGGAG GTAGGTCAAG
 251 CGGCCTTGGA AGAAGCGAGT ATCTGTTTAC TCAATTGTGG CCCTACTGGT
 301 TCCGAGGCTT TGAAGAATCT CGTACTTGGT GGTGTTGGTA GCATCACCGT
 351 TGTTGATGGA TCTAAGGTTC AATTTGGTGA CCTTGGGAAC AATTTCATGG
 401 GTACTGACTA ATTTTGAAAC TTTTTGATTG TGTTTCTTCT GGGGGTTTTG
 451 GGGGACTTGC TTGCGTTATG ATGATTTTTT TTTTTCATGT GAAGTCGATG
 501 CGAAGAGTGT TGGCCAATCA AAAGCCAAAT CTGTTTGTGC GTTTCTCCAA
 551 GAGCTTAATG ATTCTGTTAA CGCCAAGTTT ATTGAGGAGA ATCCAGACAC
 601 GTTGATTACT ACTAACCCAT CTTTCTTCTC TCAGTTCACT CTCGTTATAG
 651 CCACTCAGGT ACCATTGTTT TTCTATTTGT AATCTTCTCT AATGATTTGT
 701 GTGTTACAAT TTTTCAAAGT TCTAATCTTT GCTTACACAG TCACTCACCA
 751 TGGATCATGA TACCTTCTAT TCAATGTTCT TTAGTTAGTT TCTCATATGT
 801 ACTTTTCCTC GTCCTCTTCA CGGGATATTG CTTTATTAGG CTTCTTTTTC
 851 CTGTGTTATT GTTATTTAAG TTCGTTATTT CATCCCCTTT TCGACATTGA
 901 TTGGTTGCAG CTGGTGGAAG ATTCAATGTT GAAACTTGAT AGAATCTGTC
 951 GAGATGCAAA CGTTAAGTTG GTTTTGGTTC GCTCTTATGG CCTTGCTGGG
1001 TTTGTTCGCA TCTCTGTAAA GGTAATTAAT ACCACCTGTC TAGACCCAAT
1051 TTGTGATAAA GTTTAGATTA CTTTGTGGTA TGCCGTGGTT AGGATATTGG
1101 CCAATGAGAA TATATGCTAA GCTCTCTTCT TTTCTTTTTT ATCAGGAGCA
1151 CCCCATAATT GACTCAAAAC CTGATCATTT TCTTGACGAC CTCCGCCTGA
1201 ATAATCCATG GCCTGAACTC AAGAGGTCCA CTGCTCTCAT ATCCTCTCAC
1251 TTTATGTTGT TCTGGCATCC TTTAACATTA TGGTTGTGGA TGGACGACTG
1301 AGTACTTACT GATATTCTTA ATTATTTAAT GAACGGCAGT TTTGTGGAGA
1351 CCATTGATCT GAA**A**TGTATCA GAGCCGGCCG CTGCACATAA GCACATACCT
1401 TACGTCGTCA TTCTTGTAAA GATGGCTGAG GAGTGGGCTC AATCCCATAG
1451 TGGTAATCTT CCCTCAACCA GGGAAGAGAA AAAAGAGTTT AAGGTTTAGT
1501 TCTTTTTTGT AACTTATAAA ATTTATTAGC GAGCTTGCTT TCTTGTTTTA
1551 ATTATCTATA TCCTTGAATT CTTAGGATTT GGTTAAGTCC AAGATGGTAT
1601 CTACGGATGA AGATAACTAC AAAGAAGCCA TTGAAGCCGC TTTCAAAGTT
1651 TTTGCTCCTC GAGGAATCAG TAAGTAGATA TCTTGTTTGG TCTAGGTAGA
1701 AACTTGACTT AGAATGTTTA CTGTTTCCAT GGTTGAGTTA ATAGGTGTAG
1751 CGATTGTTGA TATTGGAGCT AAACTAACAG AAAGCTCTGT CACGGGTCAA
1801 ATCACAGTAT TCTTCATGTT GAACCAAACC TGTTTTCTCT TCCAGGCTCA
1851 GAGGTTCAAA AATTAATTAA TGATAGTTGT GCTGAAGTGA ATTCAAACTC
1901 CTCAGCTTTT TGGGTGATGG TAGCGGCTCT GAAGGTTTGA AACTTGAAAC
1951 CTGCTCTGCC AGAACTCATA ATTTTATGTT CATTTAGGAA GTGCAAACTG
2001 CAACTCATTT TGATTGAAAT CATAATCGTA CATAACCACC TGCAGGAGTT
2051 TGTTTTAAAT GAAGGTGGTG GAGAGGCACC CCTTGAAGGT TCTATACCAG
2101 ATATGACCTC TTCAACAGAG TTAGTGCACT TGCCTCTATG GCACTGAAAT
2151 TCACCCTCAG TCTATTTTTC ACATTGGTTA TGTAGCTGTA TGGTTTCTCT
2201 TTGCAGACAC TATATCAATT TGCAGAAAAT CTATTTAGCC AAAGCCGAGG
2251 CTGATTTTCT TGTCATTGAG GAACGAGTTA AAAACATTTT AAAGAAAATC
2301 GGTCGAGATC CGAGCAGCAT CCCAAAACCA ACAATCAAGA GCTTCTGCAA
2351 GAATGCAAGG AAACTTAAAG TGAGTATACT AAACTTCCTA ACATGTTTAA
2401 TTTGTTTTCT AATGTCAACG TTTTACCTAT TGCTTGTTTA CTTTTTCTTC
2451 GGAGCATTAT CAGAAACATA TGATTTGAGT AAAGCATAAA CCATAATCCG
2501 TCCTTTGTAG TTGTGCAGAT ATCGTATGGT AGAGGACGAG TTCAGAAATC
2551 CTTCTGTAAC TGAAATTCAA AAGTATTTAG CGGACGAGGA TTACAGGTGC
2601 GAATTTTCCC TTTTCATATG AGTACAAAAA CATCAGATGA TTTGAAAGAT
2651 GACCTTTCTA CAAAATGTTC ACTCTTTTGT CTGTCTCATA CATGCCATAA
2701 GATGTTGTGA TTGAATATTG CAGGAGCATT TTTTCAAAAC TGCATAATTT
2751 ACTGTATAAG CTATTCATTT AGTTGACCTG AGCTAACCAA ACTTTTTAGT
2801 TCATCTTAGG CTTACACAGC AGAAAAAATA GATTCTGGAT TTAGTTCAAA
2851 ATTTTTCTAG GCTTGCATGT TTAGCTCTTA TACTTAAATA TTGTTACTGT
2901 CGCAGTGGTG CAATGGGATT TTATATTCTT CTTAGAGCTG CGGACAGATT
2951 TGCTGCCAAC TATAACAAGT TTCCTGGGCA GTTTGATGGG TAAGAAATTC
3001 ATTATGGCAC TAAGCTTAAT CTTTATTTTT GAACAATGGG TCCTTGTTTG
3051 GCTATTAACA TGCAATACAT TTTGGTAGAG GAATGGATGA GGACATTTCT
3101 CGATTAAAAA CTACTGCCTT GAGTCTTCTT ACCGACTTGG GCTGTAACGG
3151 CTCAGTACTC CCAGATGACC TTATCCATGA GATGTGTCGC TTTGGTGCCT
3201 CAGAGATTCA TGTGGTTTCT GCCTTTGTTG GAGGAATCGC ATCTCAAGAA
3251 GTCATCAAGG TTCGATTCAT ATTTCTTTCT CATTGTTTCA AATTCAGTTA
3301 GTCTTATGGG GAAGTCGCTA GCTTCTGGAC ATGCTTAACG AGGTAATTGA
3351 ATATGTAGTT GTCTCTTTCT ATCTTTGTTT GCAGCTTGTC ACAAAGCAGT
3401 TTGTTCCGAT GTTGGGGACT TACATCTTCA ATGGCATTGA TCACAAGTCT
3451 CAGTTATTGA AATTGTAGAA GATCTTTCCT TAACACATTG GCTTGAAACA
3501 GAGAGAAAGA GCTCTATCAT ATATTATTCT TCTGATTAAA AGATAATCTT
3551 TTTGCTACTA CTGAGAAACA AAATTTTCAA TGACAGTTCA GCTGAGACCA
3601 TCCGACTCAG AATCTTCGAA AATCTTGCTT TGCTTTGTCC TTGAGGTTAG
3651 AAATATTTGA AGGTTAAATG AAGCTAATCT CATAAAATGG GCCACTGTAA
3701 GCCCATTCTA C

**Figure S1: Molecular characterisation of the *axr1* alleles**

**In EIC174**, a single nucleotide (A) insertion occurred in exon 6 (position 1364 of the genomic sequence) leading to a premature stop codon (a 222 aa protein is produced instead of 540 aa in wild type). **In EVM8**, a large in-frame deletion of 312 bp generates a 20 aa truncated protein. **In EGS344** a 907bp deletion associated with an Agrobacterium Ti plasmid DNA insertion (*) occurred in the 5’ end of the gene.
