# Supplementary figures and images for: Crossover Localisation Is Regulated by the Neddylation Posttranslational Regulatory Pathway
Source: PLoS Biol. 2014 Aug 12;12(8):e1001930. doi: 10.1371/journal.pbio.1001930 (PMC4130666; doi:10.1371/journal.pbio.1001930)

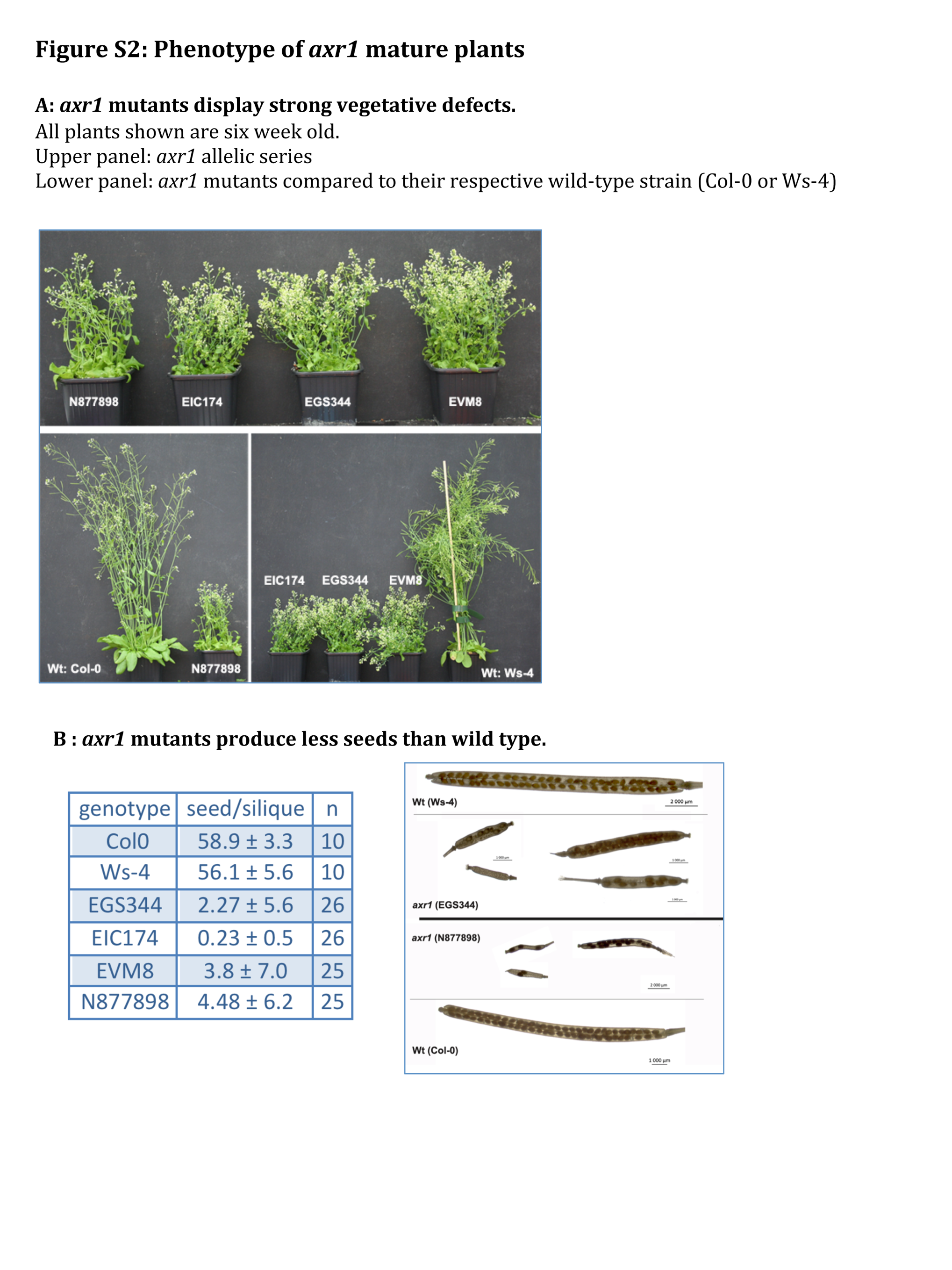

Supplement: Figure S2 — Phenotype of axr1 mature plants. (A) axr1 mutants display strong vegetative defects. All plants shown are 6 wk old. Upper panel, axr1 allelic series. Lower panel, axr1 mutants compared to their respective wild-type strain (Col-0 or Ws-4). (B) axr1 mutants produce less seeds than wild type. (TIF) [file pbio.1001930.s002.tif]

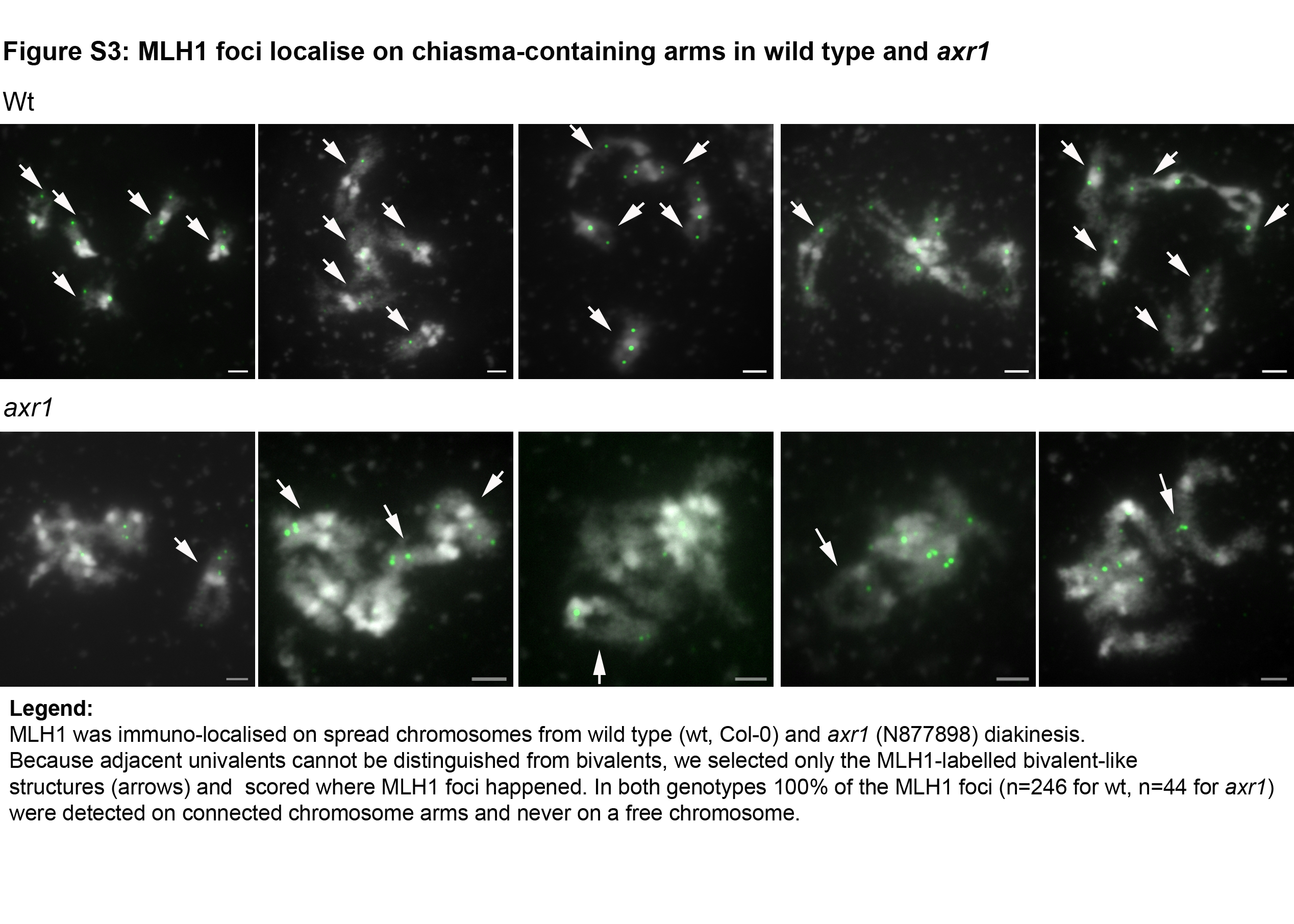

Supplement: Figure S3 — MLH1 foci localise on chiasma-containing arms in wild type and axr1. MLH1 was immunolocalised on acetic acid spread chromosomes from wild type (wt, Col-0) and axr1 (N877898) at diakinesis. Because adjacent univalents cannot be distinguished from bivalents, we selected only the MLH1-labelled bivalent-like structures (arrows) and scored where MLH1 foci occurred. In both genotypes, 100% of the MLH1 foci (n = 246 for wild type, n = 44 for axr1) were detected on connected chromosome arms and never on a free chromosome. (TIF) [file pbio.1001930.s003.tif]

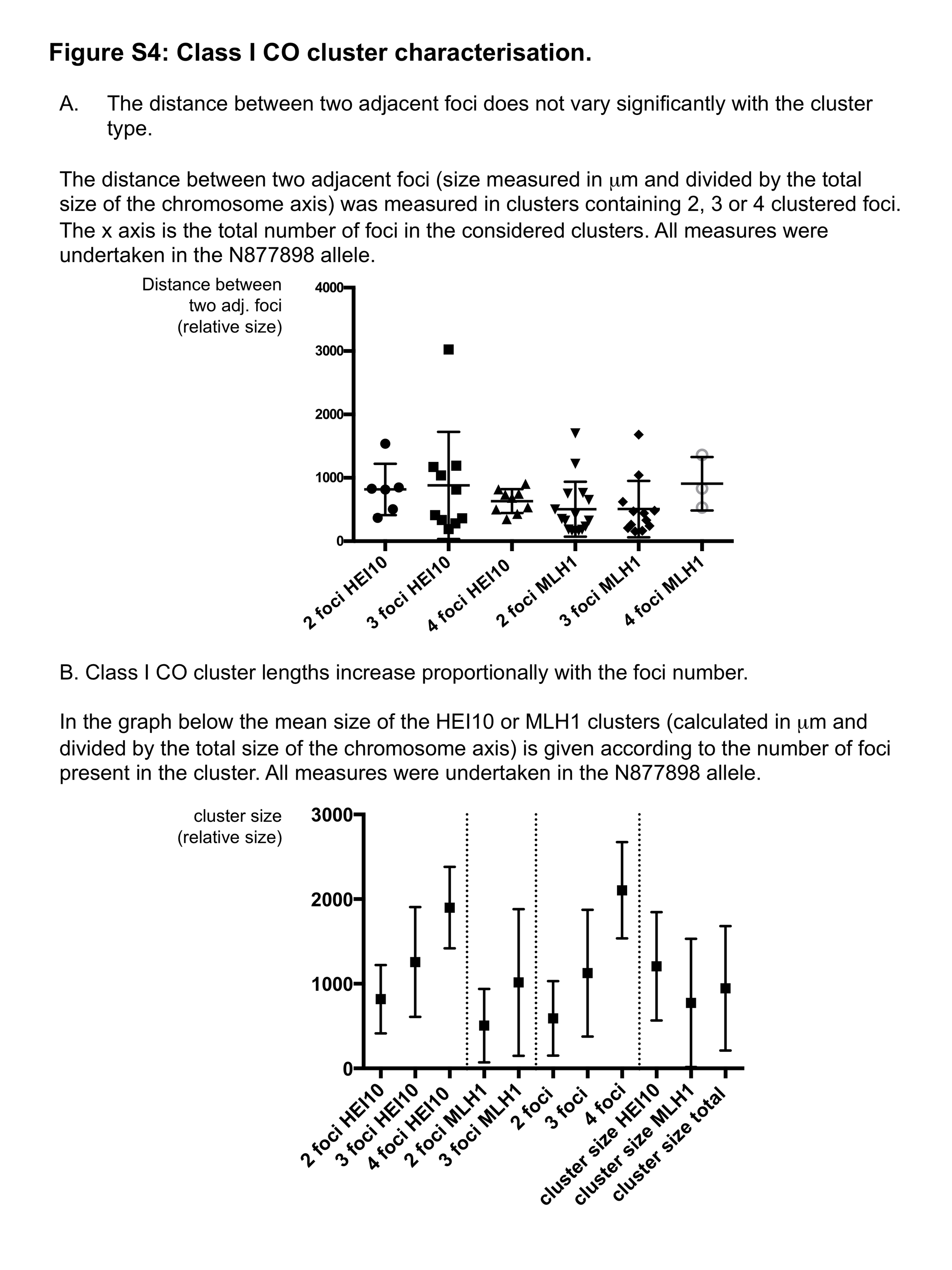

Supplement: Figure S4 — Class I CO cluster characterisation. (A) The distance between two adjacent foci does not vary significantly with the cluster type. The distance between two adjacent foci (measured in µm and divided by the total size of the chromosome axis) was measured in clusters containing two, three, or four clustered foci. The x axis is the total number of foci in the considered clusters. All measures were undertaken in the N877898 allele. (B) Class I CO cluster lengths increase proportionally with the foci number. In the graph below, the mean size of the HEI10 or MLH1 clusters (calculated in µm and divided by the total size of the chromosome axis) is given according to the number of foci present in the cluster. All measures were undertaken in the N877898 allele. (TIF) [file pbio.1001930.s004.tif]

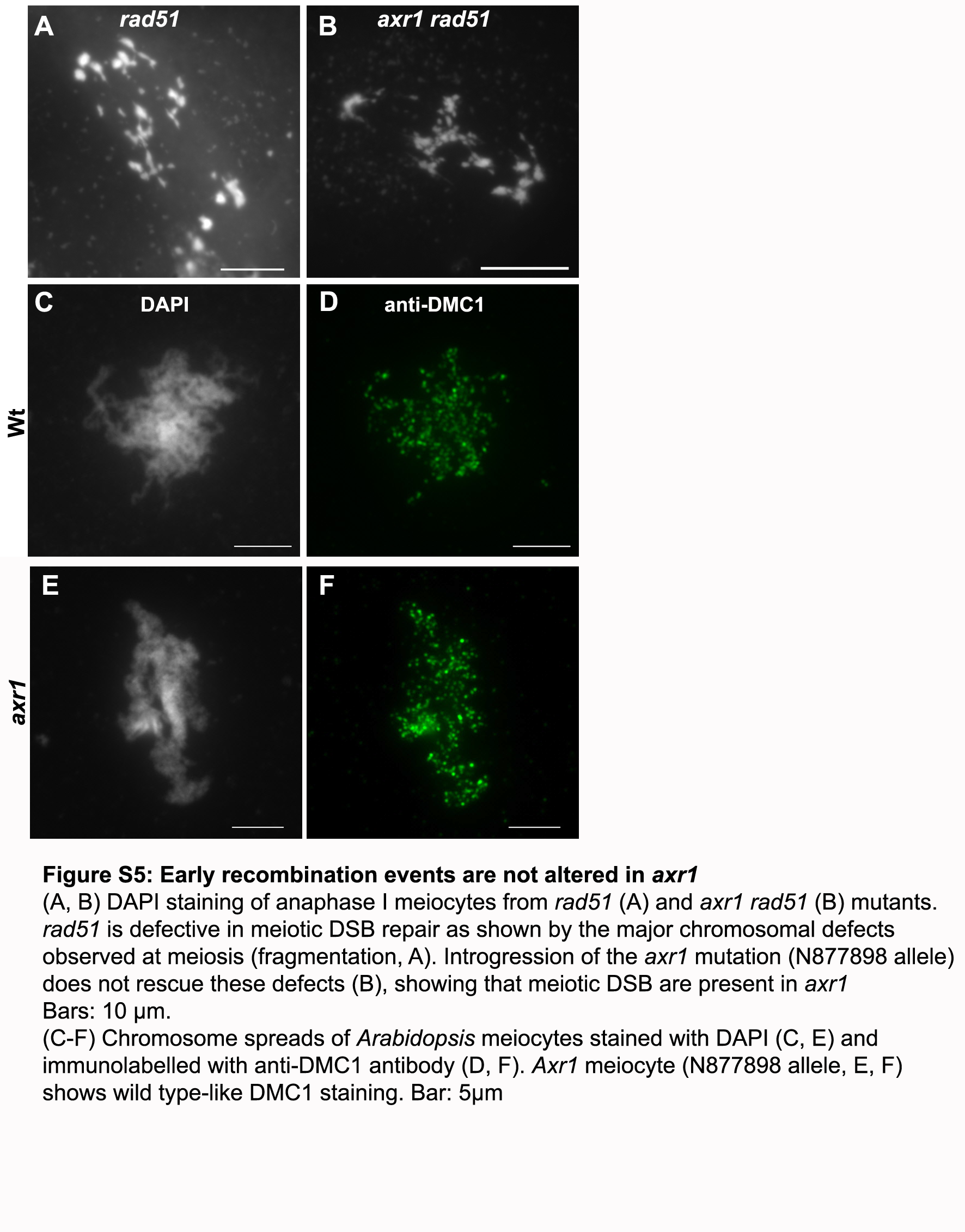

Supplement: Figure S5 — Early recombination events are not altered in axr1. (A and B) DAPI staining of anaphase I meiocytes from rad51 (A) and axr1rad51 (B) mutants. rad51 is defective in meiotic DSB repair as shown by the major chromosomal defects observed at meiosis (fragmentation, A). Introgression of the axr1 mutation (N877898 allele) does not rescue these defects (B), showing that meiotic DSB are present in axr1. Bars = 10 µm. (C–F) Lipsol chromosome spreads of Arabidopsis meiocytes stained with DAPI (C and E) and immunolabelled with the anti-DMC1 antibody (D and F). axr1 meiocytes (N877898 allele, E and F) show wild-type–like DMC1 staining. Bar = 5 µm. (TIF) [file pbio.1001930.s005.tif]

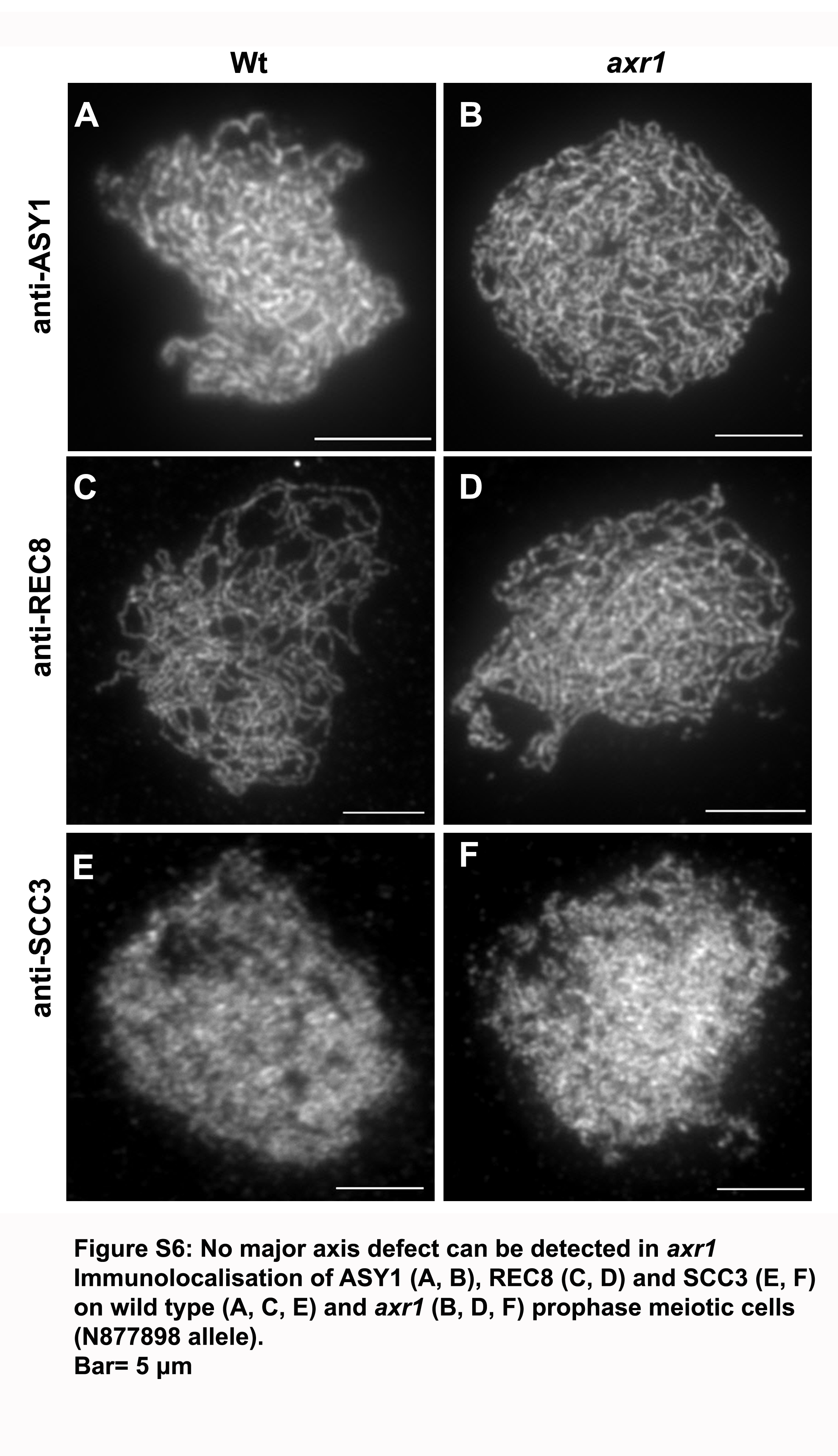

Supplement: Figure S6 — No major axis defect can be detected in axr1. Immunolocalisation of ASY1 (A and B), REC8 (C and D), and SCC3 (E and F) in wild type (A, C, and E) and axr1 (B, D, and F) prophase meiotic cells (N877898 allele). Bar = 5 µm. (TIF) [file pbio.1001930.s006.tif]

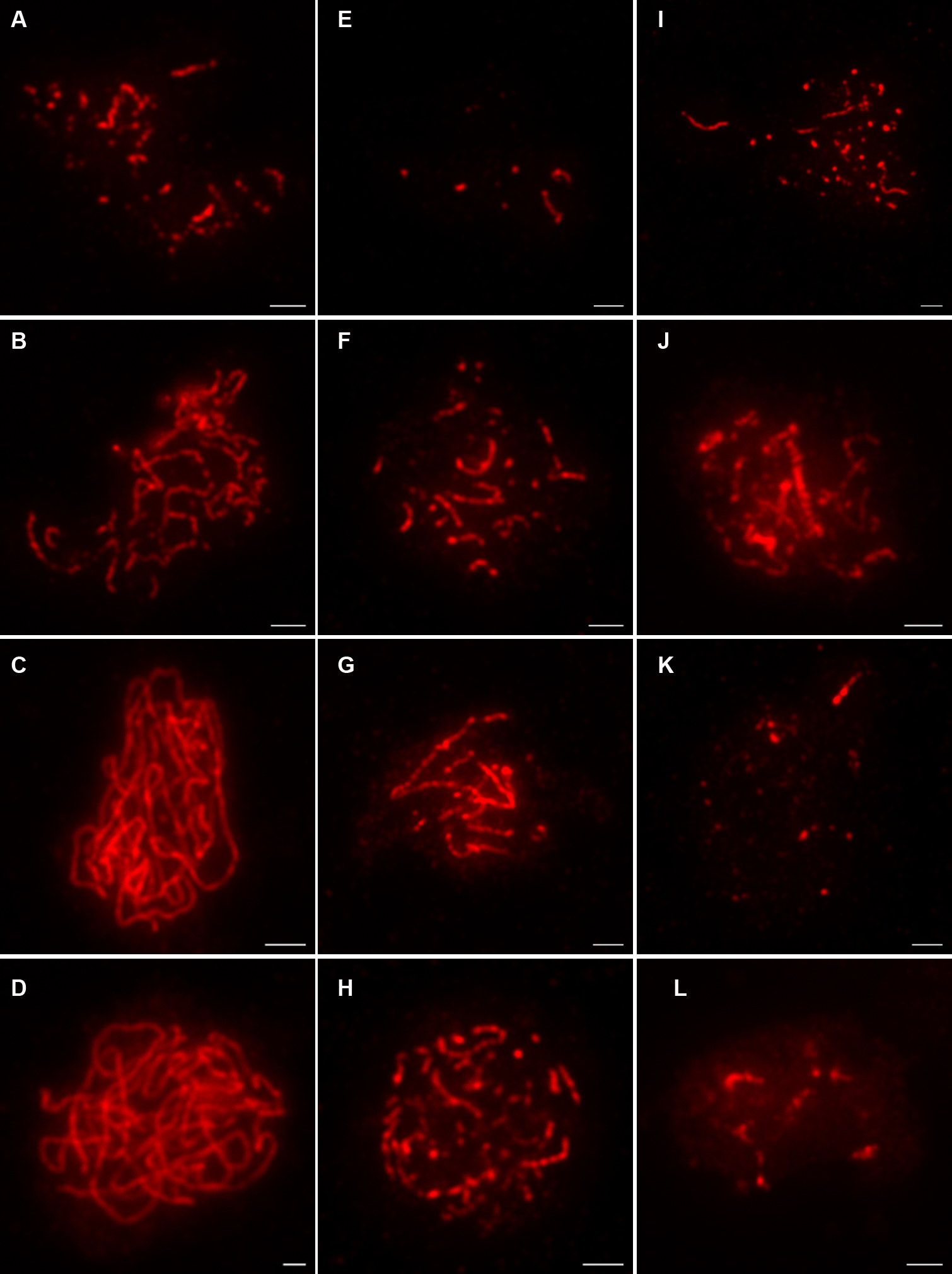

Supplement: Figure S7 — Synapsis is strongly perturbed in axr1. ZYP1 was immunolocalised on lipsol spread chromosomes from wild-type (A–D) and axr1 (N877989 allele, E–L) meiotic cells. This figure corresponds to the red channel from Figure 8. (TIF) [file pbio.1001930.s007.tif]

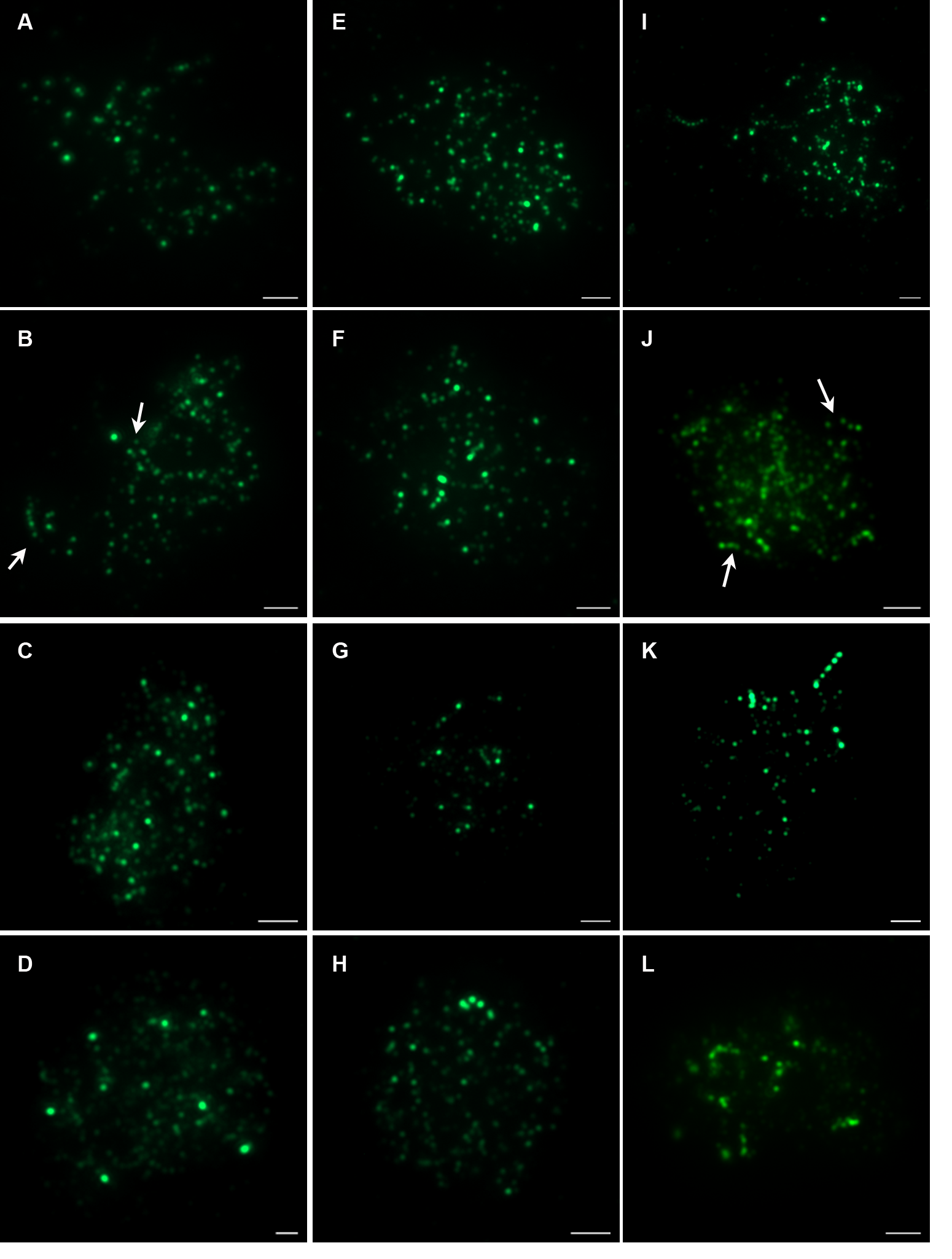

Supplement: Figure S8 — HEI10 dynamics during early prophase is unchanged in axr1. HEI10 was immunolocalised on lipsol spread chromosomes from wild-type (A–D) and axr1 (N877989 allele, E–L) meiotic cells. This figure corresponds to the green channel from Figure 8. (TIF) [file pbio.1001930.s008.tif]
