# Supplementary material for: Crossover Localisation Is Regulated by the Neddylation Posttranslational Regulatory Pathway
Source: PLoS Biol. 2014 Aug 12;12(8):e1001930. doi: 10.1371/journal.pbio.1001930 (PMC4130666; doi:10.1371/journal.pbio.1001930)
Supplement: Table S1 — Average MCN and average bivalent number per meiocyte. (DOCX) [file pbio.1001930.s009.docx]

**Table S1: Average Minimum Chiasma Number (MCN) and average bivalent number per meiocyte**

| genotype | MCN | n (MCN) | SD | bivalents | n (bivalents) | SD |
| --- | --- | --- | --- | --- | --- | --- |
| *Col-0* | 8.9 | 51 | 0.9 | 5 | 51 | 0 |
| *axr1 (N877898)* | 5.1 | 74 | 1.5 | 3.7 | 86 | 1.1 |
| *axr1 (axr1-12)* | 4.9 | 73 | 1.7 | 4.0 | 43 | 1 |
| *Ws-4* | 7.5 | 44 | 0.9 | 5 | 44 | 0 |
| *axr1 (EGS344)* | 3.1 | 55 | 1.4 | 2.7 | 73 | 1.1 |
| *axr1 (EIC174)* | 3.2 | 42 | 1.7 | 2.6 | 86 | 1.3 |
| *axr1 (EVM8)* | 2.9 | 68 | 1.7 | 2.5 | 83 | 1.3 |
| *msh5 (Col-0)* | 1.1 | 81 | 1 | 1.1 | 81 | 1 |
| *axr1zip4 (Col-0)* | 0.17 | 76 | 0.4 | 0.17 | 76 | 0.4 |
| *axr1msh5(Col-0)* | 0.18 | 67 | 0.4 | 0.18 | 67 | 0.4 |
| *axr1msh4 (Ws-4)* | 0.16 | 100 | 0.5 | 0.14 | 100 | 0.4 |
| *axr1hei10 (Ws-4)* | 0.13 | 30 | 0.3 | 0.13 | 30 | 0.3 |
| *axr1mer3(Col-0)* | 0 .57 | 58 | 0.8 | 0.55 | 58 | 0.8 |
| *axr1mlh1(Col-0)* | 0.96 | 28 | 0.7 | 2.07 | 56 | 1.2 |
| *mus81 (Col-0)* | 9.5 | 31 | 0.7 | 5 | 31 | 0 |
| *axr1mus81(Col-0)* | 5.45 | 46 | 1.6 | 3.77 | 60 | 1.0 |
| *axr1mus81msh5 (Col-0)* | 0.12 | 51 | 0.4 | 0.12 | 51 | 0.4 |
