# Supplementary material for: Crossover Localisation Is Regulated by the Neddylation Posttranslational Regulatory Pathway
Source: PLoS Biol. 2014 Aug 12;12(8):e1001930. doi: 10.1371/journal.pbio.1001930 (PMC4130666; doi:10.1371/journal.pbio.1001930)
Supplement: Table S2 — Interfoci distance within class I CO clusters. In axr1 (N877898 allele), HEI10 and MLH1 foci form clusters in approximately half of the pachytene/diplotene meiocytes. To estimate interference between adjacent foci within the clusters, we measured the distance between two adjacent foci in clusters containing more than two foci. (DOCX) [file pbio.1001930.s010.docx]

**Table S2: Inter-foci distance within class I CO clusters**

In *axr1* (N877898 allele), HEI10 and MLH1 foci form clusters in approximately half of the pachytene/diplotene meiocytes. To estimate interference between adjacent foci within the clusters, we measured the distance between two adjacent foci in clusters containing more than two foci.

| Type of foci | nb of foci in the cluster | Distance among two adjacent foci (μm) |
| --- | --- | --- |
| HEI10 | 3 | 0.95 |
|  |  | 1.21 |
|  | 3 | 0.78 |
|  |  | 1.39 |
|  | 3 | 4.2 |
|  |  | 1.66 |
|  | 3 | 2.97 |
|  |  | 7.67 |
|  | 4 | 1.42 |
|  |  | 1.21 |
|  |  | 1.94 |
|  | 4 | 3.11 |
|  |  | 2.53 |
|  |  | 2.81 |
| MLH1 | 3 | 0.5 |
|  |  | 1.55 |
|  | 3 | 1 |
|  |  | 0.8 |
|  | 3 | 1.66 |
|  |  | 1.59 |
|  | 3 | 1.29 |
|  |  | 1.02 |
|  | 3 | 6.2 |
|  |  | 3.85 |
|  | 3 | 0.68 |
|  |  | 1.24 |
|  | 4 | 1.84 |
|  |  | 1.17 |
|  |  | 3 |
