# Supplementary material for: Crossover Localisation Is Regulated by the Neddylation Posttranslational Regulatory Pathway
Source: PLoS Biol. 2014 Aug 12;12(8):e1001930. doi: 10.1371/journal.pbio.1001930 (PMC4130666; doi:10.1371/journal.pbio.1001930)

**Table S3: Recombination data set**

(A) The localisation of the six FTL intervals used in this study are shown below on the five *Arabidopsis* chromosomes. They correspond to three pairs of linked intervals I5a I5b, I5c I5d and I3b I3c described by Berchowitz and Copenhaver (Berchowitz & Copenhaver, 2008). The position (in bp) of each transgene encoding red, yellow or cyan fluorescent proteins (filled circles) is shown. The size of each interval is given in the accompanying table. The map was obtained using the Chromosome Map Tool from

http://www.arabidopsis.org/jsp/ChromosomeMap/tool.jsp

| Intervals | size (bp) |
| --- | --- |
| I5a | **4 916 298** |
| I5b | **2 650 744** |
| I5c | **1 388 133** |
| I5d | **1 736 757** |
| I3b | **2 628 078** |
| I3c | **1 192 519** |

(B) To measure recombination rates, we produced plants homozygous for the *quartet* (*qrt*) mutation, heterozygous for *axr1* (N877898 allele) and carrying or not three linked fluorescent markers (R red, Y yellow, C Cyan): *qrt*^-/-^ *axr1*^+/-^ and *qrt*^-/-^ *axr1*^+/-^ RYC/RYC. The *qrt* mutation allows the four pollen grains from a single meiosis to be maintained together. We crossed these two plants and in the progeny analysed tetrad fluorescence of mutant plants (*qrt*^-/-^ *axr1*^-/-^ RYC/+++) or fertile plants (wt) (either *qrt*^-/-^ *axr1*^+/-^ RYC/+++ or *qrt*^-/-^ *axr1*^+/+^ RYC/+++). Plants were grown in a greenhouse and tetrad analyses were carried out as described by Berchowitz and colleagues in (Berchowitz & Copenhaver, 2008). The distribution of the markers within the tetrads and the resulting distribution of colours vary depending on the number, localisation and chromatids involved in recombination. The different possibilities are indicated by drawings and the number of tetrads in each phenotypic class is shown in the tables below. Two replicates were made for the I5a I5b intervals.


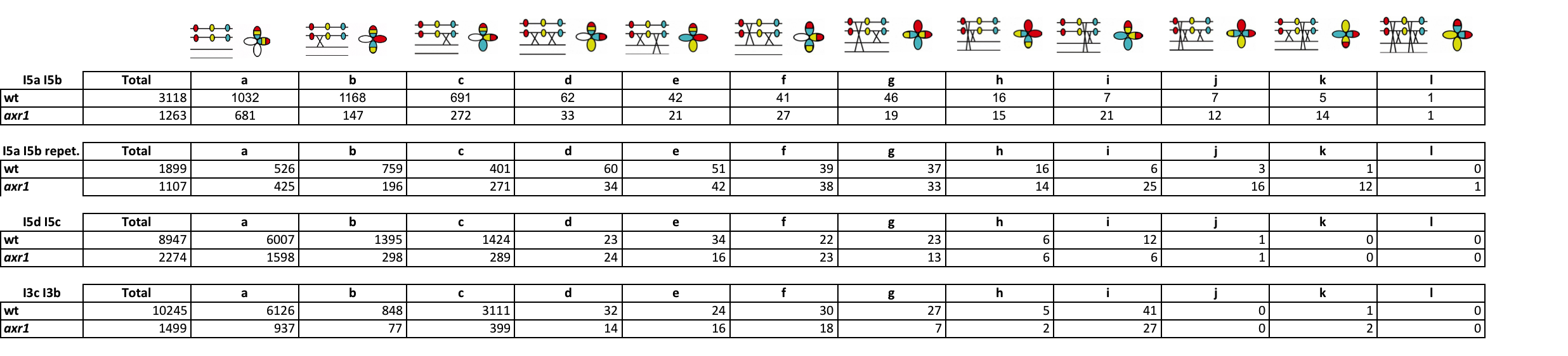

Supplement: Table S3 — Recombination dataset. (A) The localisation of the six FTL intervals used in this study are shown below on the five Arabidopsis chromosomes. They correspond to three pairs of linked intervals—I5a I5b, I5c I5d, and I3b I3c—described by Berchowitz and Copenhaver [50]. The position (in bp) of each transgene encoding red, yellow, or cyan fluorescent proteins (filled circles) is shown. The size of each interval is given in the accompanying table. The map was obtained using the Chromosome Map Tool from http://www.arabidopsis.org/jsp/ChromosomeMap/tool.jsp. (B) To measure recombination rates, we produced plants homozygous for the quartet (qrt) mutation, heterozygous for axr1 (N877898 allele), and carrying or not three linked fluorescent markers (R, red; Y, yellow; C, cyan): qrt −/− axr1 +/− and qrt −/− axr1 +/− RYC/RYC. The qrt mutation allows the four pollen grains from a single meiosis to be maintained together. We crossed these two plants and in the progeny analysed tetrad fluorescence of mutant plants (qrt −/− axr1 −/− RYC/+++) or fertile plants (wt) (either qrt −/− axr1 +/− RYC/+++ or qrt −/− axr1 +/+ RYC/+++). Plants were grown in a greenhouse, and tetrad analyses were carried out as described by Berchowitz and colleagues [50]. The distribution of the markers within the tetrads and the resulting distribution of colours vary depending on the number, localisation, and chromatids involved in recombination. The different possibilities are indicated by drawings and the number of tetrads in each phenotypic class is shown in the tables below. Two replicates were made for the I5a I5b intervals. (DOCX) [file pbio.1001930.s011.docx]
