# Supplementary material for: Crossover Localisation Is Regulated by the Neddylation Posttranslational Regulatory Pathway
Source: PLoS Biol. 2014 Aug 12;12(8):e1001930. doi: 10.1371/journal.pbio.1001930 (PMC4130666; doi:10.1371/journal.pbio.1001930)
Supplement: Tables S4 — Interinterval interference analyses. Genetic interference among COs occurring in two linked intervals was calculated using two methods: (A) The IR. The IR [18] compares the genetic size of an interval (d(Ia), in cM calculated using the Perkins equation [51]) when a CO occurs in an adjacent interval (d(Ia) with CO in Ib) to the genetic size of the same interval when no CO occurs in the adjacent interval (d(Ia) without CO in Ib). The ratio of these two distances, called the IR, gives a measurement of the strength of interference between the two intervals. When there is no interference, the ratio is equal to 1, whereas the ratio is below 1 when there is interference. Ratios above 1 indicate negative interference, indicative of more adjacent C0s than expected. We compared each IR to 1 [P(IR = 1)] and compared mutant IR to wild-type IR [P(IR = IR wt)]. Calculations and statistical analyses were performed according to Berchowitz and Copenhaver [50] using Stahl Lab Online tools (http://www.molbio.uoregon.edu/~fstahl/). In wild type, IRs were statistically below 1 for all pairs of intervals considered, indicative of interference among COs. In axr1, IRs increased systematically and were always greater than 1, but the difference with 1 was significant (p<0.01) only for intervals I5a I5b and only for one replicate of the experiment. (B) The coefficient of coincidence. The coefficient of coincidence (c.o.c.) compares the observed frequency of double COs in the two adjacent intervals [f(Ia and Ib) observed] to the expected frequency of double COs if there were no interference [f(Ia and Ib) expected]. This last frequency is the product of the frequency of COs in each single interval [f(Ia) and f(Ib)]. The c.o.c. corresponds to (f observed/f expected). When interference is absent, the c.o.c. is equal to 1. When interference is total, the c.o.c. is equal to 0. For wild type, this index varied from 0.37 to 0.63, indicative of interference. In axr1 the index was always above 1, which [file pbio.1001930.s012.docx]

**Tables S4: inter-interval Interference analyses**

Genetic interference among COs occurring in two linked intervals was calculated using two methods:

**A. The interference ratio (IR)**

The interference ratio (Malkova et al., 2004) compares the genetic size of an interval (d(Ia)*,* in cM calculated using the Perkins equation (Perkins, 1949)) when a crossover occurs in an adjacent interval (d(Ia) with CO in Ib) to the genetic size of the same interval when no crossover occurs in the adjacent interval (d(Ia) without CO in Ib). The ratio of these two distances, called the interference ratio (**IR**), gives a measurement of the strength of interference between the two intervals. When there is no interference the ratio is equal to 1, while the ratio is below 1 when there is interference. Ratios above 1 indicate negative interference, indicative of more adjacent C0s than expected.

We compared each IR to 1 (*P* (IR=1)) and compared mutant IR to wild-type IR (*P* (IR=IR wt)). Calculations and statistical analyses were performed according to Berchowitz and Copenhaver (Berchowitz & Copenhaver, 2008) using Stahl Lab Online tools (<http://www.molbio.uoregon.edu/~fstahl/>).

In wild type, IRs were statistically below 1 for all pairs of intervals considered, indicative of interference among COs. In *axr1*, IRs increased systematically and were always greater than 1, but the difference with 1 was significant (*P*<0.01) only for intervals I5a I5b and only for one replicate of the experiment.

|  |  |  |  |  |
| --- | --- | --- | --- | --- |
| **I5a I5b** | wt | *axr1* |  |  |
| d(I5a) | 24.18 | 16.98 |  |  |
| d(I5b) | 15.51 | 23.75 |  |  |
| d(I5a) without CO in I5b | 28.52 | 14.06 |  |  |
| d(I5a) with CO in I5b | 13.53 | 22.86 |  |  |
| Interference Ratio | 0.47 | 1.63 |  |  |
| *P* (IR=1) | 0 | 4 10-3 |  |  |
| *P* (IR=IRwt) | - | 8 10^-6^ |  |  |
|  |  |  |  |  |
| **I5a I5b replicate** | wt | *axr1* |  |  |
| d(I5a) | 27.94 | 24.44 |  |  |
| d(I5b) | 16.67 | 29.90 |  |  |
| d(I5a) without CO in I5b | 32.86 | 22.05 |  |  |
| d(I5a) with CO in I5b | 17.22 | 27.65 |  |  |
| Interference Ratio | 0.52 | 1.25 |  |  |
| *P* (IR=1) | 0 | 0.09 |  |  |
| *P* (IR=IRwt) | - | 10^-5^ |  |  |
|  |  |  |  |  |
| **I5d I5c** | wt | *axr1* |  |  |
| r(I5d) | 8.60 | 9.15 |  |  |
| r(I5c) | 8.94 | 8.84 |  |  |
| d(I5d) without CO in I5c | 9.66 | 8.78 |  |  |
| d(I5d) with CO in I5c | 3.51 | 11.02 |  |  |
| Interference Ratio | 0.36 | 1.26 |  |  |
| *P* (IR=1) | 0.00 | 0.11 |  |  |
| *P* (IR=IRwt) | - | 3 10^-7^ |  |  |
|  |  |  |  |  |
| **I3c I3b** | wt | *axr1* |  |  |
| r(I3c) | 4.84 | 4.87 |  |  |
| r(I3b) | 16.96 | 20.95 |  |  |
| d(I3c) without CO in I3b | 6.29 | 4.38 |  |  |
| d(I3c) with CO in I3b | 1.75 | 5.90 |  |  |
| Interference Ratio | 0.28 | 1.35 |  |  |
| *P* (IR=1) | 0.00 | 0.10 |  |  |
| *P* (IR=IRwt) | - | 1.5 10^-5^ |  |  |

**B. The coefficient of coincidence**

The coefficient of coincidence (c.o.c) compares the observed frequency of double COs in the two adjacent intervals (f(Ia and Ib) observed) to the expected frequency of double COs if there were no interference (f(Ia and Ib) expected). This last frequency is the product of the frequency of COs in each single interval (f(Ia) and f(Ib)). The coefficient of coincidence (c.o.c) corresponds to (f observed/f expected). When interference is absent, the c.o.c. is equal to 1. When interference is total, the c.o.c. is equal to 0. For wild type, this index varied from 0.37 to 0.63 indicative of interference. In *axr1,* the index was always above 1, which shows that double COs in adjacent intervals are more frequent than expected.

| **I5a I5b** | wt | *axr1* |
| --- | --- | --- |
| f(I5a) | 0.44 | 0.23 |
| f(I5b) | 0.29 | 0.33 |
| f(I5a and I5b) expected | 0.13 | 0.08 |
| f(I5a and I5b) observed | 0.06 | 0.1 |
| c.o.c. | 0.51 | 1.32 |
| *P* (c.o.c=1) | 4 10^-26^ | 1 10^-3^ |

| **I5a I5b replicate** | wt | *axr1* |
| --- | --- | --- |
| f(I5a) | 0.51 | 0.35 |
| f(I5b) | 0.31 | 0.43 |
| f(I5a and I5b) expected | 0.16 | 0.15 |
| f(I5a and I5b) observed | 0.10 | 0.16 |
| c.o.c. | 0.63 | 1.07 |
| *P* (c.o.c=1) | 1.4 10^-12^ | 0.33 |

| **I5d I5c** | wt | *axr1* |
| --- | --- | --- |
| f(I5d) | 0.17 | 0.17 |
| f(I5c) | 0.17 | 0.16 |
| f(I5d and I5c) expected | 0.03 | 0.03 |
| f(I5d and I5c) observed | 0.01 | 0.03 |
| c.o.c. | 0.40 | 1.24 |
| *P* (c.o.c=1) | 9 10^-23^ | 6 10^-2^ |

| **I3c I3b** | wt | *axr1* |  |
| --- | --- | --- | --- |
| f(I3c) | 0.09 | 0.09 |  |
| f(I3b) | 0.32 | 0.32 |  |
| f(I3c and I3b) expected | 0.03 | 0.03 |  |
| f(I3c and I3b) observed | 0.01 | 0.04 |  |
| c.o.c. | 0.37 | 1.30 |  |
| *P* (c.o.c=1) | 2.7 10^-29^ | 0.04 | |
