# Supplementary material for: Crossover Localisation Is Regulated by the Neddylation Posttranslational Regulatory Pathway
Source: PLoS Biol. 2014 Aug 12;12(8):e1001930. doi: 10.1371/journal.pbio.1001930 (PMC4130666; doi:10.1371/journal.pbio.1001930)
Supplement: Table S5 — Intra-interval interference analyses. Interference was measured within a single interval, by comparing the observed number of double COs [based on NPD frequency (NPD observed) to the expected number of double COs under the hypothesis of no interference (NPD expected)] [52]. The ratio between these two figures (NPDr) gives the strength of interference within the considered interval. The NPD tetrads correspond to h, i, j, k, and l classes from Table S2. In wild type, NPDr indicate strong interference (NPDr close to 0.3) within all the intervals (except for I3c, which is too small to give statistically meaningful figures). In axr1, NPDrs increased systematically (between 0.7 and 2.69) and were generally greater than 1, indicative of a trend toward negative interference (more double COs in a single interval than expected). However, statistical analyses on NPDr (Stahl Lab Online tools, http://www.molbio.uoregon.edu/~fstahl/) showed that these values are statistically different from 1 (p<0.01) only on I5a and I5b (one of the two replicates). (DOCX) [file pbio.1001930.s013.docx]

**Table S5: intra-interval Interference analyses**

Interference was measured within a single interval, by comparing the observed number of double COs (based on non-parental ditype (NPD) frequency (NPD observed) to the expected number of double COs under the hypothesis of no interference (NPD expected) (Stahl, 2008). The ratio between these two figures (NPDr) gives the strength of interference within the considered interval. The NPD tetrads correspond to h, i, j, k and l classes from Table S2.

In wild type, NPDr indicate strong interference (NPDr close to 0.3) within all the intervals, (except for I3c which is too small to give statistically meaningful figures). In *axr1*, NPDrs increased systematically (between 0.7 and 2.69), and were generally greater than 1, indicative of a trend towards negative interference (more double COs in a single interval than expected). However, statistical analyses on NPDr (Stahl Lab Online tools, http://www.molbio.uoregon.edu/~fstahl/) showed that these values are statistically different from 1 (p<0.01%) only on I5a and I5b (one of the two replicates).

| **I5a** | NPD observed | NPD expected | NPDr | *P* (NPDr=1) |
| --- | --- | --- | --- | --- |
| wt | 24 | 87.3 | 0.27 | 5 10^-14^ |
| *axr1* | 28 | 10.4 | 2.69 | 4 10^-8^ |
|  |  |  |  |  |
| **I5b** | NPD observed | NPD expected | NPDr | *P* (NPDr=1) |
| wt | 13 | 35.4 | 0.37 | 2 10^-4^ |
| *axr1* | 36 | 22 | 1.63 | 4 10^-3^ |

| **I5a replicate** | NPD observed | NPD expected | NPDr | *P* (NPDr=1) |
| --- | --- | --- | --- | --- |
| wt | 19 | 70.9 | 0.27 | 2 10^-12^ |
| *axr1* | 31 | 21.1 | 1.47 | 0.05 |
|  |  |  |  |  |
| **I5b replicate** | NPD observed | NPD expected | NPDr | *P* (NPDr=1) |
| wt | 7 | 25.6 | 0.27 | 3 10-4 |
| *axr1* | 38 | 32.1 | 1.18 | 0.05 |
|  |  |  |  |  |
| **I5d** | NPD observed | NPD expected | NPDr | *P* (NPDr=1) |
| wt | 7 | 32.9 | 0.21 | 10^-05^ |
| *axr1* | 7 | 8.5 | 0.82 | 0.9 |
|  |  |  |  |  |
| **I5c** | NPD observed | NPD expected | NPD ratio | *P* (NPDr=1) |
| wt | 12 | 34.6 | 0.35 | 3 10^-04^ |
| *axr1* | 6 | 8.1 | 0.74 | 0.7 |

|  |  |  |  |  |
| --- | --- | --- | --- | --- |
| **I3c** | NPD observed | NPD expected | NPDr | *P* (NPDr=1) |
| wt | 5 | 11.7 | 0.43 | 0.1 |
| *axr1* | 2 | 1.6 | 1.24 | nd |
|  |  |  |  |  |
| **I3b** | NPD observed | NPD expected | NPDr | *P* (NPDr=1) |
| wt | 42 | 141.7 | 0.30 | 4 10^-19^ |
| *axr1* | 29 | 23.3 | 1.24 | 0.4 |
