# Supplementary material for: Crossover Localisation Is Regulated by the Neddylation Posttranslational Regulatory Pathway
Source: PLoS Biol. 2014 Aug 12;12(8):e1001930. doi: 10.1371/journal.pbio.1001930 (PMC4130666; doi:10.1371/journal.pbio.1001930)
Supplement: Table S6 — Primer sequences and PCR conditions for mutant genotyping. (DOCX) [file pbio.1001930.s014.docx]

**Table S6 : Primer sequences and PCR conditions for mutant genotyping**

| *Allele* | *Line N°* | *ref.* | *wt alleles:*  *primer names*  *Tm/size* | *Primer sequences* | *mutant alleles:*  *primer names*  *Tm/size* | *Primer sequences* |
| --- | --- | --- | --- | --- | --- | --- |
| *msh4^Ws^* | EXY25 | [1] | msh4#1  msh4#2  57°/800 bp | TTATTTGTACTGCTTGGCAA  GGATCATAGAAACGCAACA | msh4#1  msh4#2  57°/750 bp | TTATTTGTACTGCTTGGCAA  GGATCATAGAAACGCAACA |
| *msh5^Col^* | SALK_026553 | [2] | N526553U N526553L  57°/750 bp | ACATGGCTTGTATTCAGCAC  CAATCAGTGTAAAACAGCAGG | N526553L  Lbsalk2  60°/750 bp | CAATCAGTGTAAAACAGCAGG  GCTTTCTTCCCTTCCTTTCTC |
| *hei10^Ws^* | EQO124 | [1] | T3F20-P5  T3F20-P6  60°/1070 bp | GGAGCAGGTCCATACAGCA  CTTATGATCCTTGTAGGTAGT | T3F20-P5  Lbsalk2  60°/970 bp | GGAGCAGGTCCATACAGCA  GCTTTCTTCCCTTCCTTTCTC |
| *zip4^Col^* | SALK_068052 | [3] | EJD21-P3R  EJD21-P5  60°/1200 bp | TCCTTCCCACACCTTGACCC  GACTGCTGGAGCAGAAACT | EJD21-P3R  Lbsalk2  60°/1200 bp | TCCTTCCCACACCTTGACCC  GCTTTCTTCCCTTCCTTTCTC |
| *mer3-2^Col^* | SALK_091560 | [4] | N591560U  N591560L  57°/1000 bp | AGCAACAAGGAAAAAGGTCAC  TACTTTGTCATCAGCCTTCCG | N591560L  Lbsalk2  60°/750 bp | TACTTTGTCATCAGCCTTCCG  GCTTTCTTCCCTTCCTTTCTC |
| *mlh1^Col^* | SK_25975 | [1] | ATGMLH1  sk25975L  57°/750 bp | ATGATCGACGATTCGTCTCTTACGG  TTGCAGATTCAATACAACAACAC | sk25975L  PSK tail 1  57°/700 bp | TTGCAGATTCAATACAACAACAC  TTCTCATCTAAGCCCCCATTTGG |
| *mus81^Col^* | SALK_107515 | [5] | N607515 U  N607515 L  60°/1000 bp | CATGCTGACAGTTGAAGGTC  CCTCAAACGTTTCTCCAAAT | N607515 L  Lbsalk2  60°/1000 bp | CCTCAAACGTTTCTCCAAAT  GCTTTCTTCCCTTCCTTTCTC |
| *rad51^Col^* | Gabi_134A01 | [6] | RAD51-fw  RAD51-rev  60°/990 bp | ATGCCAAGGTTGACAAGATTG  CTCCCCTTCCAGAGAAATCTG | RAD51-fw  LbGabi1  60°/700 bp | ATGCCAAGGTTGACAAGATTG  CCCATTTGGACGTGAATGTAGACAC |
| *mre11-4^Col^* | Salk_067823 |  | MRE11-4-P2  MRE11-4-P3  57°/890 bp | TACCACGTTTTGAAGTCCCAG  GATAGGTCCACTCGACCCAC | MRE11-4-P2  Lbsalk2  57°/1100 bp | TACCACGTTTTGAAGTCCCAG  GCTTTCTTCCCTTCCTTTCTC |
| *rmi1 ^Ws^* | FCN288 | [7] | FCN288-P2  FCN288-P7  60°/1000 bp | GCAGCTAGAGTTGCTCTGGTTG  GCTGGTCCGTTTGTTCTGCAG | FCN288-P2  LbBar2  60°/840 bp | GCAGCTAGAGTTGCTCTGGTTG  CGTGTGCCAGGTGCCCACGGAATAG |
| *cul3A* *^Col^* |  | [8] | MD35  MD139  58°/1200 bp | AGACTTCAGAGGAGACAATGCGT  GTTTCCTCCATATGTCGGAGATATCCAT | MD35  MD16  58°/1000 bp | AGACTTCAGAGGAGACAATGCGT  TGGTTCACGTAGTGGGCCATCG |
| *cul3B* *^Col^* |  | [8] | MD105  MD21  58°/670 bp | TTCTGATTCTACGATTGATCTAAGG  GCCTAGTCTGAATCTTACTCGAATAC | MD21  MD71  58°/500 bp | GCCTAGTCTGAATCTTACTCGAATAC GTGGATTGATGTGATATCTCC |
| *cul4-1 ^Col^* | Gabi_600H03 | [9] | 600H03-LP  600H03-RP  60°/1260 bp | ACGTTTTACGATATACCCCGG  GGTCCTGGAATACTCTTTCCG | 600H03-RP  LbGabi1  60°/750 bp | GGTCCTGGAATACTCTTTCCG  CCCATTTGGACGTGAATGTAGACAC |

1. Chelysheva L, Vezon D, Chambon A, Gendrot G, Pereira L, et al. (2012) The Arabidopsis HEI10 is a new ZMM protein related to Zip3. PLoS Genet 8: e1002799. Available: http://www.pubmedcentral.nih.gov/articlerender.fcgi?artid=3405992&tool=pmcentrez&rendertype=abstract. Accessed 10 July 2013.

2. Higgins JD, Vignard J, Mercier R, Pugh AG, Franklin FCH, et al. (2008) AtMSH5 partners AtMSH4 in the class I meiotic crossover pathway in Arabidopsis thaliana, but is not required for synapsis. Plant J 55: 28–39. Available: http://www.ncbi.nlm.nih.gov/pubmed/18318687. Accessed 19 November 2012.

3. Chelysheva L, Gendrot G, Vezon D, Doutriaux M-P, Mercier R, et al. (2007) Zip4/Spo22 is required for class I CO formation but not for synapsis completion in Arabidopsis thaliana. PLoS Genet 3: e83. Available: http://www.pubmedcentral.nih.gov/articlerender.fcgi?artid=1877879&tool=pmcentrez&rendertype=abstract. Accessed 10 July 2013.

4. Mercier R, Jolivet S, Vezon D, Huppe E, Chelysheva L, et al. (2005) Two meiotic crossover classes cohabit in Arabidopsis: one is dependent on MER3,whereas the other one is not. Curr Biol 15: 692–701. Available: http://www.ncbi.nlm.nih.gov/pubmed/15854901. Accessed 4 June 2013.

5. Berchowitz LE, Francis KE, Bey AL, Copenhaver GP (2007) The role of AtMUS81 in interference-insensitive crossovers in A. thaliana. PLoS Genet 3: e132. Available: http://www.pubmedcentral.nih.gov/articlerender.fcgi?artid=1941751&tool=pmcentrez&rendertype=abstract. Accessed 19 November 2012.

6. Li W, Chen C, Markmann-Mulisch U, Timofejeva L, Schmelzer E, et al. (2004) The Arabidopsis AtRAD51 gene is dispensable for vegetative development but required for meiosis. Proc Natl Acad Sci U S A 101: 10596–10601. Available: http://www.pubmedcentral.nih.gov/articlerender.fcgi?artid=489980&tool=pmcentrez&rendertype=abstract.

7. Chelysheva L, Vezon D, Belcram K, Gendrot G, Grelon M (2008) The Arabidopsis BLAP75/Rmi1 homologue plays crucial roles in meiotic double-strand break repair. PLoS Genet 4: e1000309. Available: http://www.pubmedcentral.nih.gov/articlerender.fcgi?artid=2588655&tool=pmcentrez&rendertype=abstract. Accessed 10 July 2013.

8. Thomann A, Lechner E, Hansen M, Dumbliauskas E, Parmentier Y, et al. (2009) Arabidopsis CULLIN3 genes regulate primary root growth and patterning by ethylene-dependent and -independent mechanisms. PLoS Genet 5: e1000328. Available: http://www.pubmedcentral.nih.gov/articlerender.fcgi?artid=2607017&tool=pmcentrez&rendertype=abstract. Accessed 5 September 2013.

9. Bernhardt A, Lechner E, Hano P, Schade V, Dieterle M, et al. (2006) CUL4 associates with DDB1 and DET1 and its downregulation affects diverse aspects of development in Arabidopsis thaliana. Plant J 47: 591–603. Available: http://www.ncbi.nlm.nih.gov/pubmed/16792691. Accessed 3 September 2013.
